# Impaired Mitochondrial Fusion and Oxidative Phosphorylation Triggered by High Glucose Is Mediated by Tom22 in Endothelial Cells

**DOI:** 10.1155/2019/4508762

**Published:** 2019-05-19

**Authors:** Yi Zeng, Qi Pan, Xiaoxia Wang, Dongxiao Li, Yajun Lin, Fuli Man, Fei Xiao, Lixin Guo

**Affiliations:** ^1^PeKing University Fifth School of Clinical Medicine, Beijing, China; ^2^Department of Endocrinology, Beijing Hospital, National Center of Gerontology, Beijing, China; ^3^The MOH Key Laboratory of Geriatrics, Beijing Hospital, National Center of Gerontology, Beijing, China

## Abstract

Much evidence demonstrates that mitochondrial dysfunction plays a crucial role in the pathogenesis of vascular complications of diabetes. However, the signaling pathways through which hyperglycemia leads to mitochondrial dysfunction of endothelial cells are not fully understood. Here, we treated human umbilical vein endothelial cells (HUVECs) with high glucose and examined the role of translocase of mitochondrial outer membrane (Tom) 22 on mitochondrial dynamics and cellular function. Impaired Tom22 expression and protein expression of oxidative phosphorylation (OXPHOS) as well as decreased mitochondrial fusion were observed in HUVECs treated with high glucose. The deletion of Tom22 resulted in reduced mitochondrial fusion and ATP production and increased apoptosis in HUVECs. The overexpression of Tom22 restored the balance of mitochondrial dynamics and OXPHOS disrupted by high glucose. Importantly, we found that Tom22 modulates mitochondrial dynamics and OXPHOS by interacting with mitofusin (Mfn) 1. Taken together, our findings demonstrate for the first time that Tom22 is a novel regulator of both mitochondrial dynamics and bioenergetic function and contributes to cell survival following high-glucose exposure.

## 1. Introduction

Vascular complications are the main cause of disability and death in patients with diabetes [1]. Endothelial dysfunction may be an initiating factor and key mechanism of atherogenesis [2, 3]. An improved understanding of the mechanisms underlying endothelial dysfunction could stimulate new approaches for the prevention and management of diabetic cardiovascular disease.

Mitochondria are essential for endothelial cell survival and function, and mitochondrial dysfunction plays a critical role in atherogenesis. Human mitochondria contain 1500 different proteins, 99% of which are encoded by nuclear DNA [4]. In addition, all mitochondrial preproteins are recognized and imported by a central entry gate: the translocase of outer mitochondrial membrane (Tom) complex [5, 6]. The Tom complex comprises seven components: the central channel protein, Tom40; the receptor proteins (Tom20, Tom22, and Tom70); and three small proteins that modulate the stability of the complex (Tom5, Tom6, and Tom7) [7, 8]. Tom20 and Tom70 initially recognize the precursor proteins and transfer them to the central receptor Tom22 and from there to the Tom40 import channel [9]. Accumulating evidence shows that the components of the Tom complex exhibit plasticity in response to various stressors, including pathological cardiac hypertrophy [10], cytosolic kinases, and increased levels of thyroid hormone [11]. These studies indicate that the Tom complex might incorporate a molecular switch that fine-tunes mitochondrial biogenesis and bioenergetics to meet metabolic demands under stress.

Tom22 not only acts as an important mitochondrial receptor but also is involved in the assembly of the Tom complex [12, 13]. Studies have revealed that Tom22 is essential for cell viability; in yeast, disruption of Tom22 strongly reduced growth and the import of mitochondrial proteins [12]. In addition, in the absence of Tom22, the import machinery lacks tight control of channel gating [12] and imported proteins are inactive due to misfolding [14]. Gerbeth et al. reported that protein kinases involved in glucose-induced signal transduction, including casein kinase 1, casein kinase 2 (CK2), and protein kinase A (PKA), could regulate the import and assembly of Tom22 in yeast [15]. Thus, determining whether Tom22 plays a critical role in diabetes and vascular complications has been of great interest.

Mitochondria are plastic organelles that frequently change their morphology, number, and intracellular distribution in response to fluctuations in metabolic demands. The disruption of this delicate balance results in altered mitochondrial fragmentation, mitochondrial membrane potential (MMP) heterogeneity, and apoptosis [16–18]. Mitochondrial fusion is regulated by mitofusin (Mfn) 1 and 2 and optic atrophy-1 (Opa1). Proteins controlling fission include dynamin-related protein-1 (Drp1), fission-1 (Fis1), and mitochondrial fission factor (Mff). Recent work has highlighted the importance of mitochondrial dynamics in diabetes [19] and endothelial cell dysfunction [20, 21]. Studies have implicated mitochondrial fission as a key mediator of the decrease in ATP production and cellular apoptosis under hyperglycemic conditions [22]. In addition, OXPHOS is responsible for the production of ATP inside mitochondria and is thus a vital factor in cell metabolism. However, the mechanisms that regulate the alterations in mitochondrial dynamics and OXPHOS in diabetes, as well as the relationship between mitochondrial dynamics and OXPHOS, remain to be explored.

In the present study, we report that Tom22 plays a critical role in the high-glucose-induced dysfunction of human umbilical vein endothelial cells (HUVECs) by decreasing mitochondrial fusion and OXPHOS. The overexpression of Tom22 reverses endothelial cell dysfunction induced by high glucose by restoring the balance of mitochondrial dynamics and OXPHOS. Furthermore, Tom22 regulates mitochondrial fusion and fission through its interaction with the mitochondrial fusion protein Mfn1. Increasing expression of Mfn1 also could reverse changes in mitochondrial dynamics and OXPHOS under high-glucose conditions. To our knowledge, this study is the first to show a link between Tom22 and endothelial dysfunction.

## 2. Materials and Methods

### 2.1. Confocal Microscopy and Quantification Analysis of Mitochondrial Morphology

Mitochondrial morphology was visualized by confocal microscopy. HUVECs were plated in confocal dishes. The cells were incubated with 100 nmol/l MitoTracker® Deep Red (Thermo Fisher, USA) for 30 min at 37°C, washed with PBS three times, and fixed with 4% paraformaldehyde for 15 min. Multiple *z*-stack section images were acquired with an Eclipse Ti confocal microscope (Nikon, Japan) using a 60x/1.25 oil immersion objective.

For the quantitative analysis of mitochondrial morphology, the acquired images were analyzed with ImageJ (National Institutes of Health, USA) according to [23] for the calculation of the form factor (FF, the reciprocal of the circularity value) and aspect ratio (AR, the ratio of the length of the major axis to the length of the minor axis) [22, 23]. Both FF and AR have a minimal value of 1 when a particle is a small, perfect circle, and the values of each increase as the shape becomes elongated. Specifically, the AR is a measure of mitochondrial length, and an increase in the FF represents an increase in both mitochondrial length and branching. Volume reconstitution of *z*-stacks of thresholded images was performed. The mitochondrial volume of per cell was quantified with the ImageJ 3D Object Counter plug-in (NIH, USA). Each experiment was performed at least three times, and 16–25 cells per condition were quantified.

### 2.2. Western Blotting

HUVECs were lysed in radioimmunoprecipitation buffer (Beyotime, China) containing a protein inhibitor cocktail (Sigma, USA). Proteins were separated by SDS-PAGE, transferred to polyvinylidene fluoride membranes, and blocked with 5% nonfat milk in PBS at room temperature for 2 hr. Membranes were incubated with the appropriate primary antibody at 4°C overnight followed by an HRP-conjugated secondary antibody for 1 hr at room temperature. Protein levels were normalized to that of *β*-tubulin. The immunoblots were developed with Western Lightning ECL detection reagent (Millipore, USA).

Mouse monoclonal antibodies against Tom22 (Abcam, UK) diluted at 1 : 300, Mfn1 (Abcam, UK) diluted at 1 : 300, tubulin (Proteintech, USA) diluted at 1 : 2000, Mfn2 (Proteintech, USA) diluted at 1 : 1000, Drp1 (Proteintech, USA) diluted at 1 : 1600, p-Drp1 (Ser616, Affinity, USA) diluted at 1 : 500, Opa1 (Abcam, UK) diluted at 1 : 1000, Fis1 (Proteintech, USA) diluted at 1 : 1000, and Mff (Proteintech, USA) diluted at 1 : 1000 were used to assess the relative protein expression levels and physical interactions. NADH dehydrogenase (ubiquinone) Fe-S protein 1 (NDFUS1, Proteintech, USA) diluted at 1 : 1000; Cytochrome c oxidase subunit 2 (mtCOx2, Proteintech, USA) diluted at 1 : 1000; and succinate dehydrogenase complex, subunit B (SDHB, Proteintech, USA), diluted at 1 : 1000 were used to evaluate the oxidative phosphorylation.

### 2.3. Real-Time PCR

Total mRNA was extracted using TRIzol reagent (Invitrogen, USA) according to the manufacturer's protocol. Complementary DNA (cDNA) was synthesized using a PrimeScript™ RT reagent kit (Takara, China). Real-time PCR was carried out using a SYBR® PrimeScript™ RT-PCR kit (Takara, China). Samples were subjected to the following protocol for mRNA detection: 5 min at 95°C; 40 cycles of 30 s at 95°C, 30 s at 60°C, and 30 s at 72°C; and a final step of 10 min at 72°C and 5 min at 4°C.

The amplification primers used were as follows: Tom22: forward, 5′-gacagcaggagctcatttcc-3′ and reverse, 5′-Ttgggcctatggctgttaag-3′; Mfn1: forward, 5′-ttggagcggagacttagcat-3′ and reverse, 5′-ttcgatcaagttccggattc-3′; and tubulin: forward, 5′-tctgcacggtctctttgtct-3′ and reverse, 5′-ccagagaagccatgacagga-3′. SDHB: forward, 5′-ggaaggcaagcagcagtatc-3′ and reverse, 5′-agcgataggcctgcataaga-3′. NDUFS1: forward, 5′-gccgatttttagaggggaag-3′ and reverse, 5′-caaacctgatgcagcgagta-3′. Cox2: forward, 5′-gaatcattcaccagtgtgcc-3′ and reverse, 5′-tggcagggttgctggtggtagga-3′. The data were analyzed using the 2^-ΔΔCT^ method.

### 2.4. HUVEC Culture

Umbilical cords were obtained from the maternity ward of the Beijing Hospital. HUVECs were extracted with collagenase (1 mg/ml) digestion. The Research Ethics Committee of the Beijing Hospital approved the collection. HUVECs were cultured in endothelial cell medium (ScienCell Research Laboratories, USA) at 37°C in a humidified atmosphere of 5% CO_2_. Cells were used at passage 2–3. For high glucose treatment, cells were exposed to culture medium with 15 mmol/l or 30 mmol/l D-glucose for 24 or 48 hr.

### 2.5. ATP Levels

Cellular ATP levels were detected using a luminescence-based ATP assay kit (Beyotime, China) according to the manufacturer's instructions. Briefly, HUVECs were lysed with 100 *μ*l ATP detection lysis buffer in 12-well plates and centrifuged at 12,000 × *g* for 5 min. Ten microliters of each supernatant was mixed with 100 *μ*l ATP working solution and measured using a Synergy H1 microplate reader (BioTek, USA). The ATP release levels were expressed as a percentage of the luminescence levels in the treated control cells [24]. For statistical analysis, the experiments were repeated at least three times, with *n* = 3 per treatment condition. The ATP concentration was normalized to the cell density for each sample.

### 2.6. Apoptosis Assay

To assess apoptosis, HUVECs were examined by using a FITC Annexin V Apoptosis Detection Kit with propidium iodide (PI) (BioLegend, USA). HUVECs were double-stained with Annexin V-FITC and PI according to the manufacturer's instructions, and apoptotic cells were the detected with an Accuri C6 flow cytometer [25] (BD, USA).

### 2.7. Mitochondrial Membrane Potential

For MMP detection, HUVECs were harvested with 0.25% trypsin-EDTA and resuspended in 0.5 ml culture medium at approximately 2 × 10^6^ cells/ml, followed by incubation with 0.5 ml JC1 working solution (Beyotime, China) at 37°C and 5% CO_2_ for 20 min. For the positive control condition, 10 *μ*M CCCP was used to pretreat the cells at 37°C for 20 min. The cells were washed twice with a staining buffer and detected using the Accuri C6 flow cytometer (BD, USA). The ratio between Rhodamine and FITC channel fluorescence intensity was used as index of MMP. MMP% was the MMP of treated cells divided by that of control cells and multiplied by 100%.

### 2.8. Transmission Electron Microscopy

HUVECs were washed twice with PBS, with the plates gently scraped off in the presence of PBS, and transferred to 1.5 ml Eppendorf tubes. After centrifugation at 1000 rpm for 5 min, the HUVECs were fixed in a 0.1 M sodium cacodylate buffer (pH 7.4) containing 2.5% glutaraldehyde overnight at 4°C and were then postfixed in 0.1 M osmium tetroxide at room temperature for 2 h. The specimens were dehydrated with a graded series of ethanol and embedded in epoxide resin. Ultrathin sections were placed on formvar/carbon grids and were stained with uranyl acetate and lead citrate. The sections were analyzed using a TEM-1400Plus transmission electron microscope (JEOL, Japan).

### 2.9. Lentiviral Infection and Small Interfering RNA (siRNA) Transfection

Recombinant lentiviral vectors expressing HBLV-Tom22-flag-PURO and HBLV-Mfn1-PURO were created at Hanbio Biotechnology Co Ltd. (China). In addition, the HBLV-PURO vector was used as a negative control. HUVECs were cultured and infected with lentivirus at a multiplicity of infection (MOI) of 50.

The siRNAs were created at GenePharma (China) and were transfected according to the manufacturer's instructions. We used three pairs of siRNA to knock down the expression of Tom22. The effect of siRNA 342 was most significant and was used for subsequent experiments. The sequences were as follows: Tom22 siRNA 287 sense (5′-3′): GGAUUGGGACCACUUCCUUTT, antisense (5′-3′): AAGGAAGUGGUCCCAAUCCTT; 342 sense (5′-3′): GAAGUUGCAAAUGGAGCAATT, antisense (5′-3′): UUGCUCCAUUUGCAACUUCTT; 437 sense (5′-3′): CCUCACUUCCUGGAAAGAUTT, antisense (5′-3′): AUCUUUCCAGGAAGUGAGGTT. We used three pairs of siRNA to knock down the expression of Mfn1. The effect of siRNA 982 was most obvious and was used for subsequent experiments. Mfn1 siRNA 440 sense (5′-3′): GGCCAUAUAACCAAUUGCUTT, antisense (5′-3′): AGCAAUUGGUUAUAUGGCCTT; 982 sense (5′-3′): GGAAGUUCUUAGUGCUAGATT, antisense (5′-3′): UCUAGCACUAAGAACUUCCTT; 1836 sense (5′-3′): CGCCAGAUAAUGCAUCACATT, antisense (5′-3′): UGUGAUGCAUUAUCUGGCGTT. We used three pairs of siRNA to knock down the expression of Drp1. The effect of siRNA 2083 was most significant and was used for subsequent experiments. The sequences were as follows: Drp1 siRNA 791 sense (5′-3′): CCCUAGCUGUAAUCACUAATT, antisense (5′-3′): UUAGUGAUUACAGCUAGGGTT; 1162 sense (5′-3′): CUCCAACUUAUUACCAAAUTT, antisense (5′-3′): AUUUGGUAAUAAGUUGGAGTT; 2083 sense (5′-3′): CAGCGAGAUUGUGAGGUUATT, antisense (5′-3′): UAACCUCACAAUCUCGCUGTT.

### 2.10. Coimmunoprecipitation (CO-IP)

Cells were lysed in RIPA buffer [50 mM Tris (pH 7.4), 150 mM NaCl, 1% Triton X-100, 1% sodium deoxycholate, and 0.1% SDS] supplemented with a protein inhibitor cocktail. Endogenous Tom22 was immunoprecipitated from total extracts with a specific antibody (Abcam, UK) and protein A/G agarose (Thermo Fisher, USA). After extensive washing with RIPA buffer containing 300 mM NaCl, the immunocomplexes were denatured by boiling, electrophoresed by SDS/PAGE, and transferred to polyvinylidene fluoride membranes. FIS1, Mff, Mnf1, Opa1, and Tom22 were visualized by incubation with specific antibodies, appropriate HRP-conjugated secondary antibodies, and standard chemiluminescence reagents (Millipore, USA). The whole lysate was used as the positive control. For the negative controls, the assay was performed using a nonspecific antibody from the same species as the IP antibody.

### 2.11. Mitochondrial DNA Quantitation

For mitochondrial DNA (mtDNA) quantification, we used a primer set that detects a relatively stable site in mitochondrial DNA minimal arc: mtDNA F: CTAAATAGCCCACACGTTCCC; R: AGAGCTCCCGTGAGTGGTTA; single-copy nuclear DNA was quantified with the beta-2M gene: nuclear DNA F: GCTGGGTAGCTCTAAACAATGTATTCA; R: CCATGTACTAACAAATGTCTAAAATGGT [26]. Relative mitochondrial DNA copy number was calculated as the difference in threshold amplification between mtDNA and nuclear DNA [26].

### 2.12. ROS

DCFH-DA was diluted to 1 : 1000 with serum-free medium to a final concentration of 10 *μ*mol/l. The cells were collected and suspended in diluted DCFH-DA at a concentration of one million/ml and incubated in a 37°C cell incubator for 20 minutes. The cells were washed three times with serum-free cell culture medium to sufficiently remove DCFH-DA that did not enter the cells. Samples were detected with a fluorescence microplate reader. ROS% was expressed as the ROS of treated cells divided by that of control cells and multiplied by 100%.

### 2.13. Statistical Analyses

The data are presented as the means ± SEMs. The data are all independent experiments. Statistical significance was assessed by performing analysis of variance (ANOVA) followed by the Tukey-Kramer post hoc analysis for multiple comparisons. *P* < 0.05 was considered statistically significant. Statistics were computed with GraphPad Prism software (GraphPad Software, USA).

## 3. Results

### 3.1. Impairment of Tom22 Expression and the Mitochondrial Network in HUVECs Treated with High Glucose

**Figure 1:**
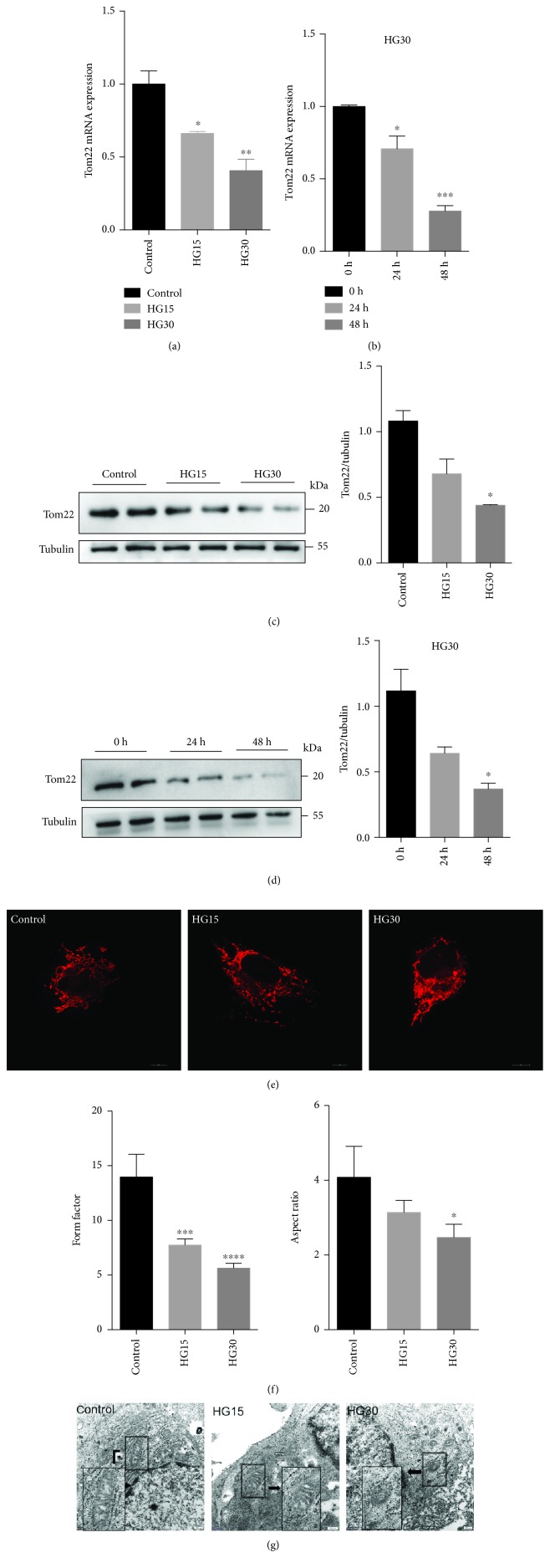
Impairment of Tom22 expression and the mitochondrial network of HUVECs treated with high glucose. (a) Real-time PCR analysis of Tom22 expression in HUVECs exposed to different glucose concentrations for 48 hr (*n* = 3). (b) Real-time PCR analysis of Tom22 expression in HUVECs exposed to 30 mmol/l D-glucose for 24 or 48 hr (*n* = 3). (c) Representative Western blot and quantification analysis of the Tom22 (22 kDa) protein level in HUVECs exposed to different concentrations of glucose for 48 hr (*n* = 6). (d) Representative Western blot and quantification analysis of the Tom22 (22 kDa) protein level in HUVECs exposed to 30 mmol/l D-glucose for 24 or 48 hr (*n* = 6). (e) Representative confocal microscopy images of HUVECs stained with MitoTracker® Deep Red at different concentrations for 48 hr. Scale bar: 10 *μ*m. (f) Quantification of the form factor (FF) and aspect ratio (AR) of the mitochondrial networks seen in (e) (*n* = 4). (g) Representative transmission electron microscopy images. Most mitochondria became smaller and punctate with significantly short in length in HUVECs treated with high glucose. Scale bar: 200 nm. (^∗^ *P* < 0.05, ^∗∗^ *P* < 0.01, ^∗∗∗^ *P* < 0.001, and ^∗∗∗∗^ *P* < 0.0001 vs. control). Control: normal culture medium; HG15: D-glucose 15 mmol/l; and HG30: D-glucose 30 mmol/l. All data are presented as the mean ± SEMs.

To investigate the underlying role of Tom22 in hyperglycemia, we measured the mRNA and protein levels of Tom22, which might undergo changes in HUVECs exposed to high glucose. The real-time PCR analysis revealed that the mRNA levels of Tom22 significantly decreased in HUVECs as the concentration of glucose increased (Figure 1(a)). Furthermore, the expression of Tom22 mRNA significantly decreased as the duration of incubation in high glucose increased (Figure 1(b)). The expression of the Tom22 protein was also downregulated progressively in a glucose dose-dependent and time-dependent manner (Figures 1(c) and 1(d)). In addition, high glucose did not affect the protein levels of Tom20, Tom40, and Tom70 (Figure S1A). The mRNA and protein levels of Tom22 in HUVECs treated with mannitol at concentrations of 15 mmol/l and 30 mmol/l were not different from those in the control cells (data not shown).

To determine the changes in the mitochondrial network, we examined the mitochondrial morphology in HUVECs using confocal fluorescence microscopy and electron transmission microscopy. Mitochondria in HUVECs under normal conditions appeared as long tubular structures in interconnected networks. Upon the shift to high-glucose conditions, mitochondria were fragmented into short rods or spheres (Figure 1(e)). The confocal quantification analysis showed that high glucose significantly decreased both the FF and the AR of mitochondria in HUVECs in a dose-dependent pattern (Figure 1(f)). Furthermore, mitochondrial ultrastructure exhibited increased fragmentation and shortened with increasing glucose concentration, as observed by transmission electron microscopy (Figure 1(g)). In normal conditions, mitochondria showed a long filamentous morphology in HUVECs. In contrast, most mitochondria became smaller and punctate with significantly shorter lengths in HUVECs treated with high glucose (Figure 1(g)). These changes were suggestive of mitochondrial fragmentation.

### 3.2. Effect of High Glucose on Genes Related to Mitochondrial Dynamics and OXPHOS of HUVECs

**Figure 2:**
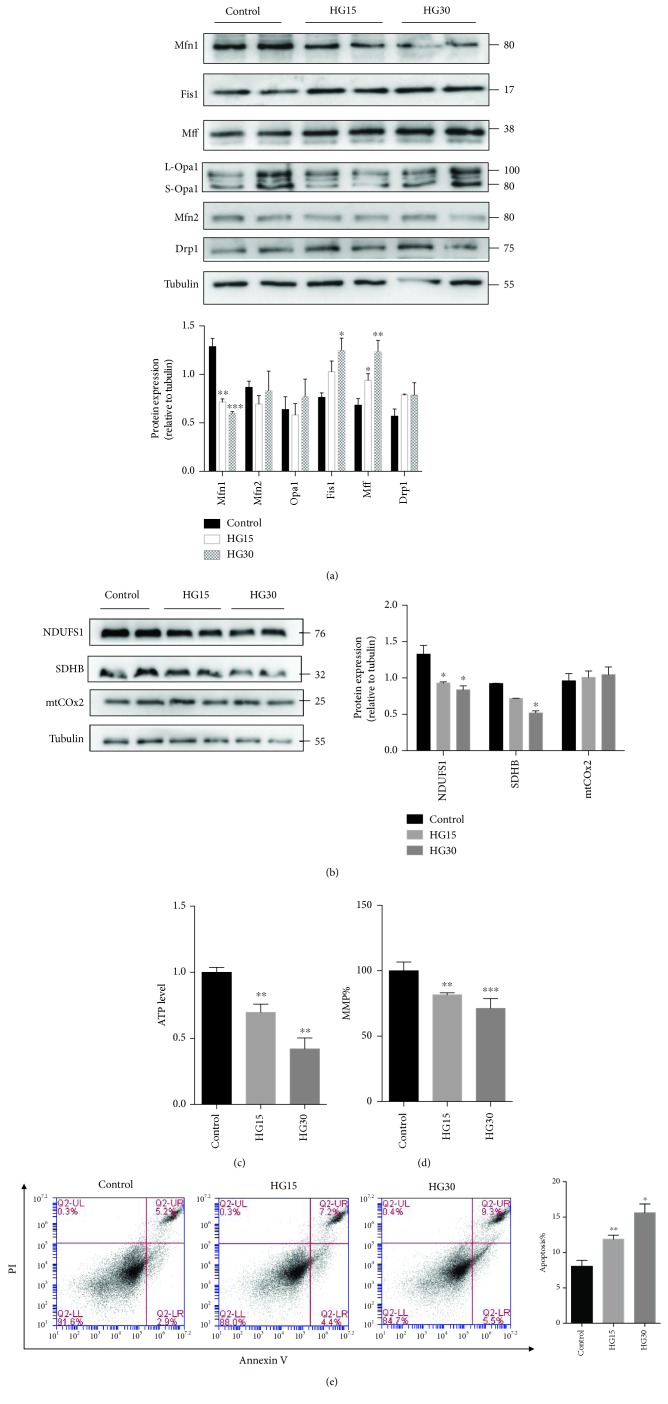
Effect of high glucose on the expression of genes regulating mitochondrial dynamics and mitochondrial function. (a) Representative Western blot and quantification analysis of the level of proteins regulating mitochondrial dynamics in each group. The expression of Fis1 and Mff increased significantly upon treatment with high glucose, whereas the expression of Mfn1 decreased significantly. No significant change was seen in the level of Mfn2, Opa1, or Drp1 (*n* = 4). (b) Representative Western blot and quantification analysis of the level of proteins that are involved in OXPHOS; the loading control was *β*-tubulin (*n* = 4). (c) Total ATP level in HUVECs exposed to different concentrations of glucose for 48 hr (*n* = 4). (d) MMP was analyzed by staining with JC1 and was quantified based on fluorescence intensities. MMP: mitochondrial membrane potential (*n* = 3). (e) Apoptotic cells were detected at 48 h by Annexin V/PI staining and flow cytometric analysis (*n* = 4) (^∗^ *P* < 0.05, ^∗∗^ *P* < 0.01, and ^∗∗∗^ *P* < 0.001 vs. control). Control: normal culture medium; HG15: D-glucose 15 mmol/l; and HG30: D-glucose 30 mmol/l. All data are presented as the means ± SEMs.

We then detected the expression of proteins encoded by genes that regulate mitochondrial fusion and fission. Western blotting revealed a significant decrease in the level of the Mfn1 protein and a significant increase in the levels of Fis1, p-Drp1 (phospho-Drp1), and Mff in HUVECs after treatment with high glucose (Figure 2(a) and Figure S1B). However, the levels of the Mfn2 and L-Opa1/S-Opa1 proteins were unchanged (Figure 2(a) and Figure S1C). Moreover, we examined the expression of genes in respiratory chain enzyme complexes I (NDUFS1), complex II (SDHB), and complex IV (mtCOx2) using QPCR and western bolting. The mRNA levels and protein levels of NDUFS1 and SDHB were significantly decreased in HUVECs exposed to high glucose (Figure S1D and Figure 2(b)). High glucose did not affect the expression of mtCOx2 in HUVECs (Figure S1D and Figure 2(b)).

To analyze whether hyperglycemia regulates mitochondrial function, we detected ATP production and the MMP. Compared to normal glucose conditions, high glucose conditions resulted in a dose-dependent decrease in both the ATP production (Figure 2(c)) and the MMP (Figure 2(d)). Furthermore, apoptosis increased significantly as the glucose concentration increased (Figure 2(e)). The mtDNA copy number was not changed significantly in HUVECs exposed to high glucose (30 mmol/l) compared with that under normal conditions (Figure S1E). Mitochondrial volume was significantly decreased in HUVECs exposed to high glucose (Figure S1F). We found that high glucose significantly increased the ROS levels of HUVECs (Figure S1G).

### 3.3. Tom22 Downregulation Results in Decreased Mitochondrial Fusion and OXPHOS and Increased Cell Apoptosis

**Figure 3:**
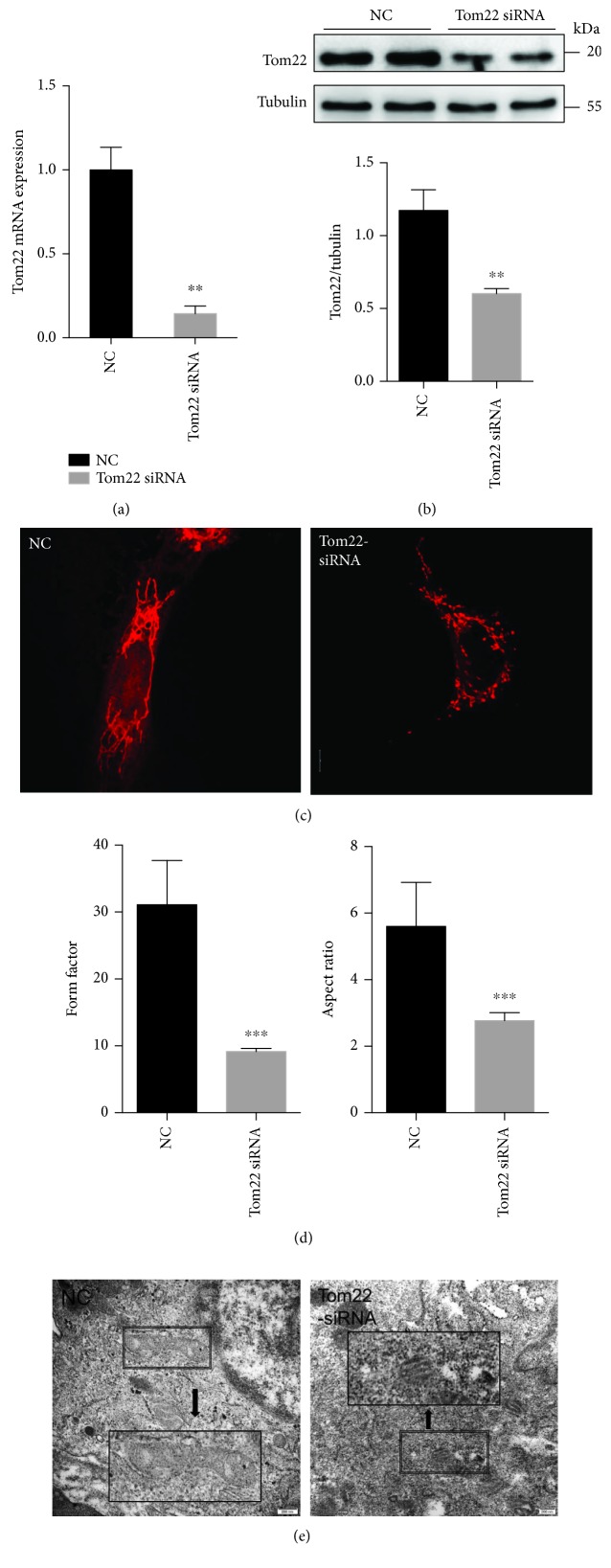
Loss of Tom22 shifts the balance of mitochondrial morphology toward fission. HUVECs were transfected with Tom22 siRNA to knock down the expression of Tom22. NC represents the negative control. (a) The real-time PCR analysis showed that Tom22 expression was decreased by siRNA knockdown (*n* = 3). (b) Western blot analysis was detected to evaluate the efficiency of the Tom22 siRNA knockdown (*n* = 4). (c) Representative confocal microscopy images of HUVECs stained with MitoTracker® Deep Red. Scale bar: 10 *μ*m. (d) Quantification of the form factor and aspect ratio of the mitochondrial networks (*n* = 4). (e) Representative images of mitochondria visualized by transmission electron microscopy. Mitochondria shortened in length in the Tom22 knockdown group. Scale bar: 200 nm (^∗^ *P* < 0.05, ^∗∗^ *P* < 0.01, and ^∗∗∗^ *P* < 0.001 vs. NC). NC: HUVECs transfected with negative control siRNA; Tom22 siRNA: HUVECs transfected with Tom22 siRNA to knock down the expression of Tom22. All data are presented as means ± SEMs.

To confirm the link between Tom22 downregulation and HUVEC dysfunction, we knocked down the expression of Tom22 by siRNA. At the mRNA level, a significant reduction in Tom22 expression of 85% was seen after siRNA knockdown of Tom22 (Figure 3(a)); the downregulation of Tom22 at the protein level was also confirmed (Figure 3(b)). There was no significant difference in protein levels of Tom20, Tom40, and Tom70 between the Tom22 knockdown group and the NC group (Figure S2A). With Tom22 knockdown, mitochondrial fragmentation increased, as shown via MitoTracker staining (Figure 3(c)); the quantification analysis showed that the FF and AR decreased significantly in the Tom22 knockdown cells compared with those in the negative control cells (Figure 3(d)). Electron transmission microscopy further confirmed that mitochondria shortened in length in the Tom22 knockdown group (Figure 3(e)). These results indicate that Tom22 is essential for the maintenance of mitochondrial morphology and that the knockdown of Tom22 expression shifted mitochondrial dynamics toward fission.

**Figure 4:**
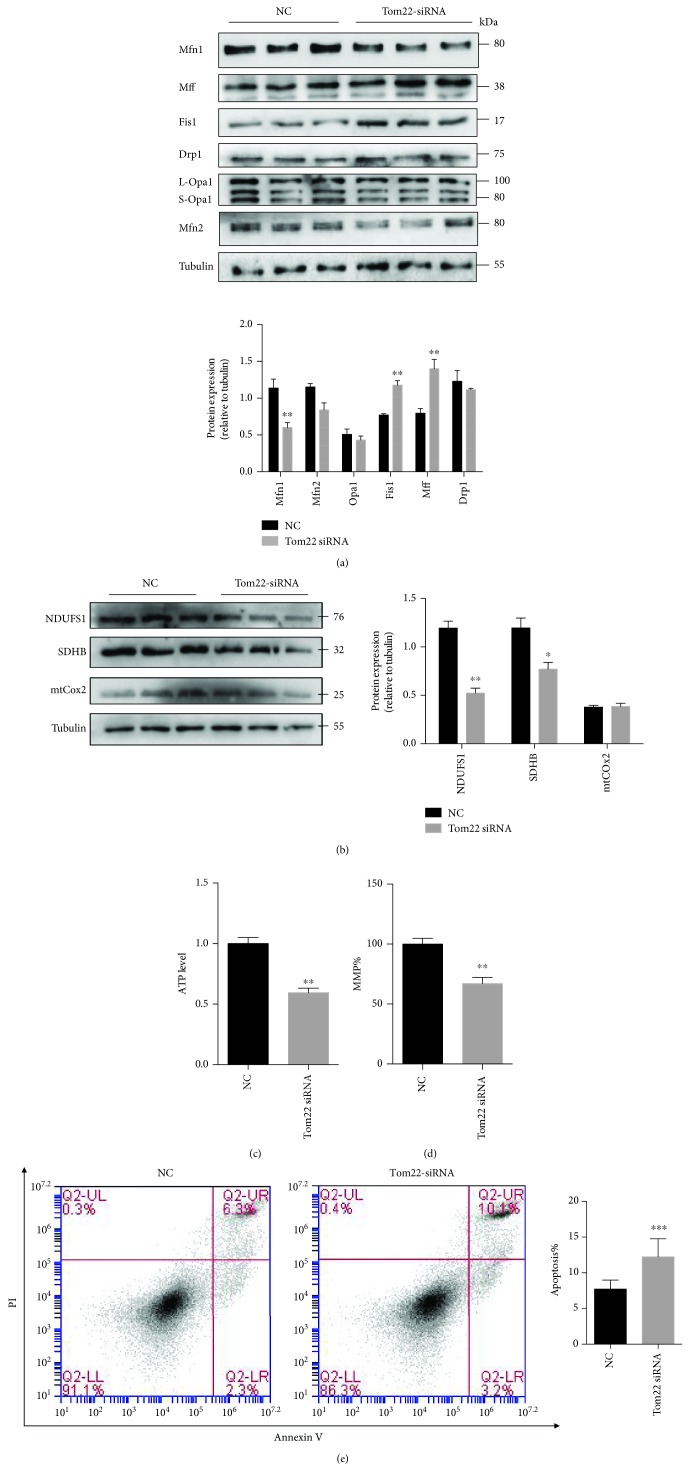
Effects of Tom22 downregulation on the expression of genes regulating mitochondrial dynamics and on apoptosis in HUVECs. (a) Western blot and quantification analysis of the expression of proteins regulating mitochondrial dynamics. The expression of Mfn1 decreased significantly, but the expression of both Fis1 and Mff increased significantly. No significant change in the level of Mfn2, Opa1, or Drp1 was found (*n* = 3). (b) Representative Western blot and quantification analysis of the level of proteins that involve in OXPHOS (*n* = 4). (c) The total ATP level was significantly decreased with Tom22 knockdown (*n* = 4). (d) The MMP was significantly decreased in HUVECs with Tom22 knockdown (*n* = 3). (e) Apoptosis was significantly increased after Tom22 knockdown (*n* = 4). (^∗^ *P* < 0.05, ^∗∗^ *P* < 0.01, and ^∗∗∗^ *P* < 0.001 vs. NC). NC: negative control; Tom22 siRNA: HUVECs transfected with Tom22 siRNA to knock down the expression of Tom22. All data are presented as means ± SEMs.

To obtain further insight into the molecular mechanism underlying the regulation of mitochondrial fusion and fission by Tom22, we evaluated the expression of mitochondrial dynamics-related genes. The Mfn1 protein level was significantly lower in the Tom22 knockdown cells than in the negative control cells (Figure 4(a)). The protein expression of the Fis1, Mff, and p-Drp1 was significantly higher in the Tom22 knockdown cells than in the control cells. Furthermore, the levels of other proteins involved in the regulation of mitochondrial dynamics, including L-Opa1/S-Opa1, Mfn2, and Drp1, were unchanged (Figure 4(a) and Figure S2C). Downregulation of Tom22 significantly decreased the mRNA levels and protein levels of NDUFS1 and SDHB, which are involved in OXPHOS (Figure 2SD and Figure 4(b)). There was no significant difference in expression of mtCOx2 between the two groups (Figure 2SD and Figure 4(b)).

In addition, Tom22 knockdown decreased the ATP production (Figure 4(c)) and MMP (Figure 4(d)) in HUVECs. Apoptosis was markedly increased in the Tom22 knockdown cells compared to that in the control cells (Figure 4(e)). Knockdown of Tom22 did not significantly affect the mtDNA copy number (Figure S2E). Mitochondrial volume was reduced when Tom22 was downregulation (Figure S2F).

### 3.4. Overexpression of Tom22 Protects HUVECs from High-Glucose-Induced Mitochondrial Fragmentation and Apoptosis

**Figure 5:**
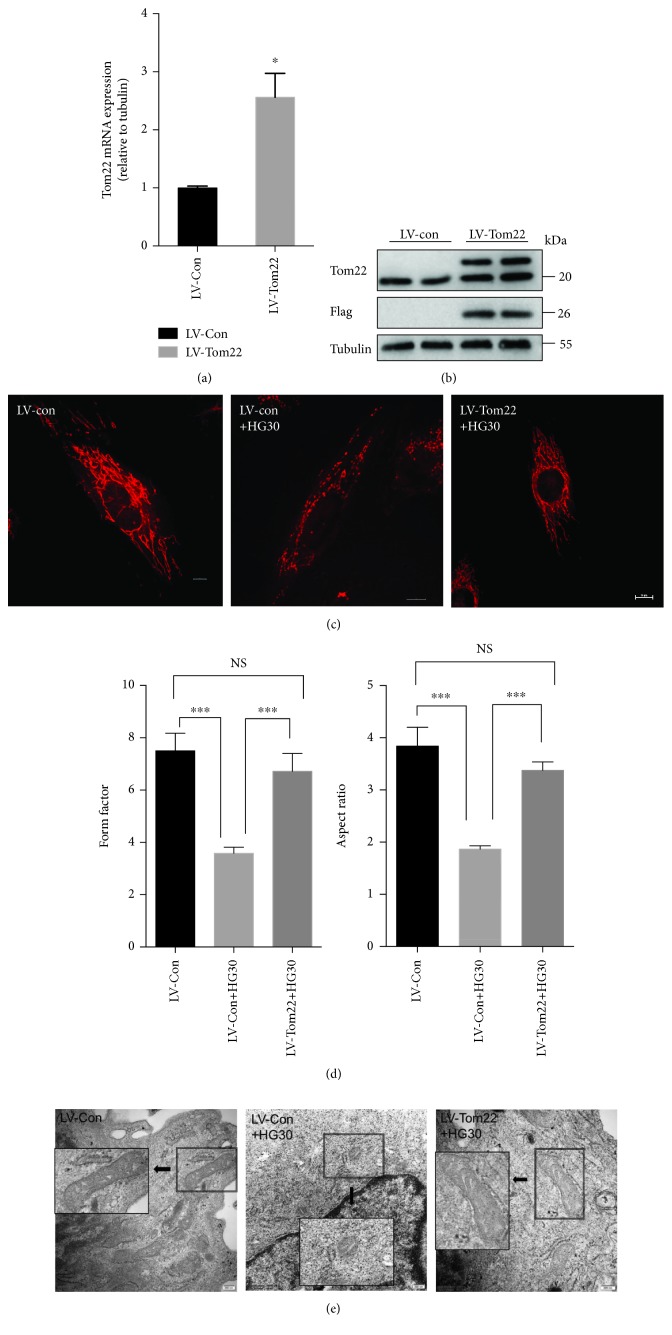
Tom22 overexpression restored mitochondrial morphology. HUVECs were transduced with LV-Tom22 vectors that expressed Tom22. The LV-Con vector was used as a vehicle control. (a) The overexpression of Tom22 was confirmed by real-time PCR (*n* = 3). (b) A Western blot analysis was conducted to evaluate the overexpression efficiency of LV-Tom22 (*n* = 3). (c) Cells were stained with MitoTracker. Scale bar: 10 *μ*m. (d) Quantification of the form factor and aspect ratio of the mitochondrial networks (*n* = 4). (e) Representative images of mitochondria visualized by transmission electron microscopy. When Tom22 overexpression, mitochondria morphology returned to longer which shortened by high glucose. Scale bar: 200 nm (^∗^ *P* < 0.05, ^∗∗^ *P* < 0.01, and ^∗∗∗^ *P* < 0.001 vs. LV-Con; NS = not significant) All data are presented as means ± SEMs.

To confirm the role of Tom22 in mediating high-glucose-induced mitochondrial dysregulation in HUVECs, we subsequently examined whether the overexpression of Tom22 might inherently protect against the pathological processes induced by high glucose. To this end, HUVECs were infected with lentivirus to overexpress Tom22. The real-time PCR and western blot analyses confirmed the overexpression of Tom22 (Figure 5(a), Figure 5(b), and Figure S3C). Protein levels of Tom20, Tom40, and Tom70 did not change significantly when TOM22 was overexpressed (Figure S3A).

The mitochondria appeared as short rods and round fragments in the LV-Con+HG30 group. The mitochondria in the LV-Tom22+HG30 group exhibited long tubular networks, which indicated that the reversal of the high-glucose-induced reduction in the Tom22 level reestablished homeostasis of mitochondrial fusion and fission events in HUVECs (Figure 5(c)). The mitochondria in the LV-Tom22+HG30 group exhibited a higher FF and AR than did the mitochondria in the LV-Con+HG30 group (Figure 5(d)). Similar trends in mitochondrial morphology changes were confirmed by electron transmission microscopy. When Tom22 was overexpressed, mitochondria returned to a longer morphology, which was shortened by high glucose (Figure 5(e)).

**Figure 6:**
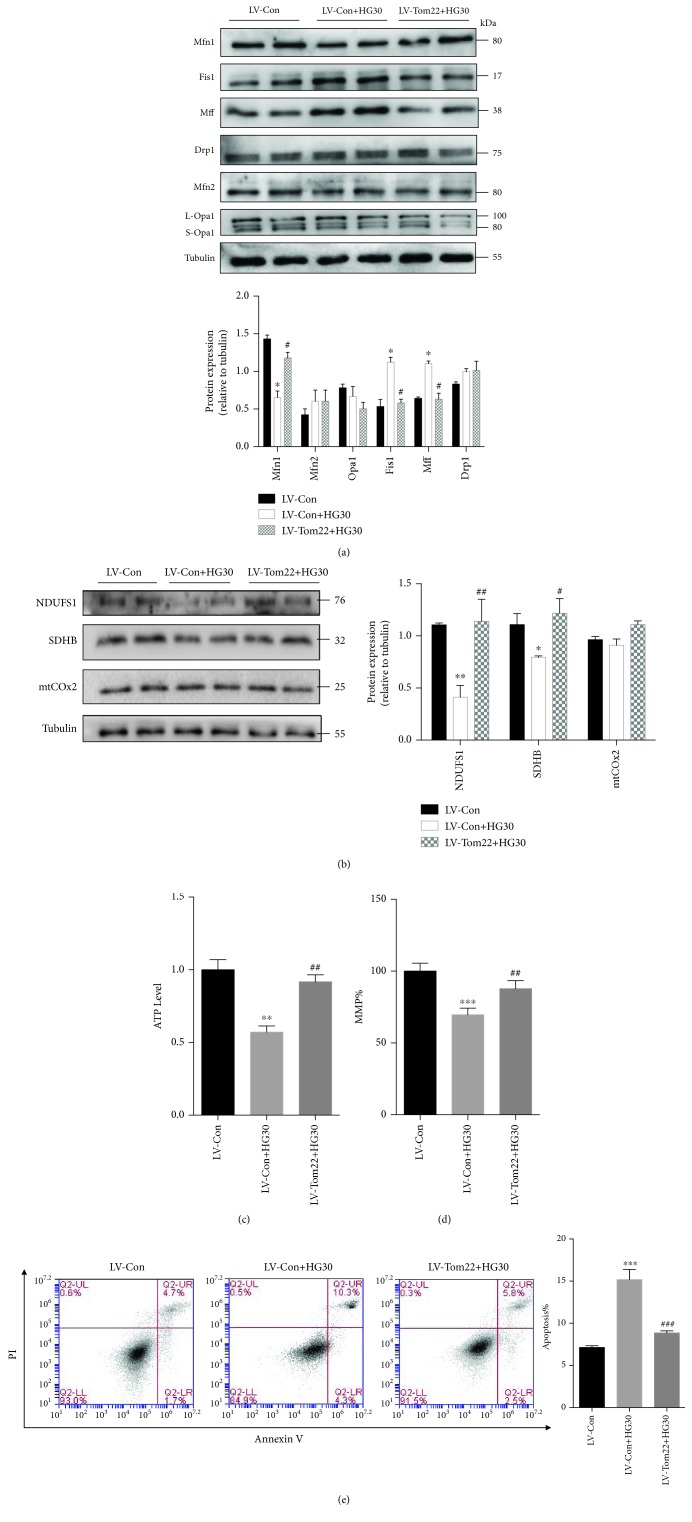
Tom22 overexpression attenuated the mitochondrial dysfunction and apoptosis induced by high glucose. HUVECs were transduced with LV-Tom22 vectors that expressed Tom22. The LV-Con vector was used as a vehicle control. (a) Western blot and quantification analysis of the level of proteins regulating mitochondrial dynamics. The expression of Mfn1 decreased significantly, and the expression of both Fis1 and Mff increased significantly. No significant change in the expression of Mfn2, Opa1, or Drp1 was seen (*n* = 4). (b) Representative Western blot and quantification analysis of the level of proteins that involve in OXPHOS (*n* = 4). (c) Total ATP level in HUVECs (*n* = 4). (d) The MMP was analyzed by JC1 staining and quantified based on fluorescence intensities (*n* = 3). (e) Apoptotic cells were detected by Annexin V/PI staining (*n* = 3). All data are presented as means ± SEMs (^∗^ *P* < 0.05, ^∗∗^ *P* < 0.01, and ^∗∗∗^ *P* < 0.001 vs. LV-Con; ^#^ *P* < 0.05 and ^##^ *P* < 0.01 vs. LV-Con+HG30).

To further investigate the molecular mechanisms underlying the Tom22-mediated reduction in mitochondrial fusion, the expression of proteins related to mitochondrial dynamics was assessed. Following high glucose exposure, the Mfn1 protein level was reduced in the LV-Con group. The level of Mfn1 was increased under high-glucose conditions after Tom22 overexpression compared with that in the LV-Con group (Figure 6(a)). Overexpression of Tom22 significantly decreased the levels of Fis1, p-Drp1, and Mff that were increased under high-glucose conditions (Figure 6(a) and Figure S3B). In addition, mRNA and protein levels of NDUFS1 and SDHB were significantly increased in the LV-Tom22 group under high glucose compared with their levels in the LV-Con group (Figure 6(b) and Figure S3E). There was no significant difference in protein expression of Drp1, L-Opa1/S-Opa1, Mfn2, and mtCOx2 between the LV-Tom22 and LV-Con groups under the high glucose conditions (Figure 6(a) and Figure 6(b) and Figure S3D).

Moreover, the ATP assay results demonstrated that Tom22 overexpression ameliorated the injury to the respiratory function of mitochondria induced by high glucose (Figure 6(c)), and Tom22 overexpression significantly attenuated the high-glucose-induced loss of MMP and increase in apoptosis (Figure 6(d) and Figure 6(e)). There was no significant difference in mtDNA copy number between the LV-Con group and the LV-Tom22 group (Figure S3F). Mitochondrial volume was significantly increased in the LV-Tom22 group than that of the LV-Con group under the 30 mM glucose condition (Figure S3G). Overexpression of Tom22 could restore the ROS levels that were increased by high glucose (Figure S3H).

### 3.5. Tom22 Binds to Mfn1

**Figure 7:**
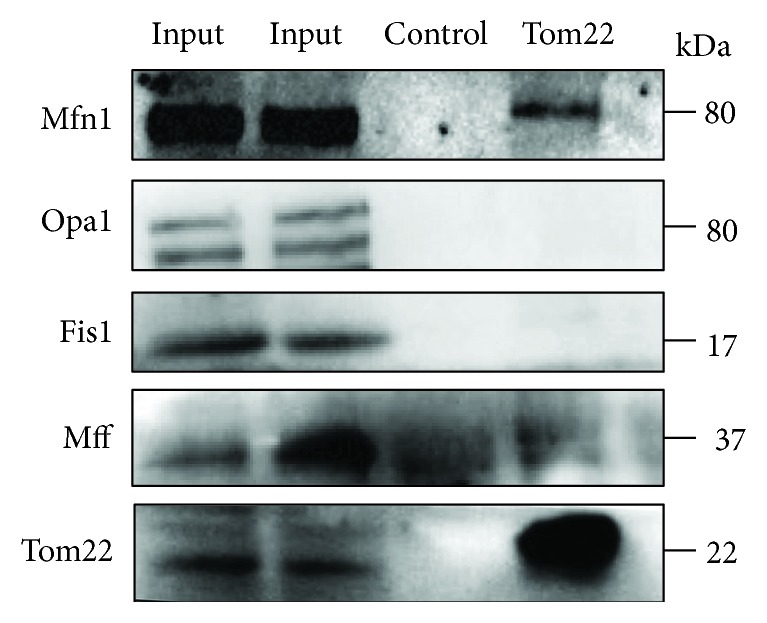
Tom22 binds to Mfn1. Coimmunoprecipitation of Tom22 with Mfn1, Fis1, Mff, and Opa1 in HUVECs.

We further assessed the potential associations between Tom22 and proteins that involve mitochondrial dynamics using a CO-IP assay. The CO-IP experiment demonstrated that Tom22 interacts physically with MFN1 but not with either Fis1 or Mff (Figure 7). These data demonstrated that Tom22 binds with Mfn1 directly. Moreover, we quantified the mRNA levels of Mfn1 via real-time PCR. We observed that the Mfn1 transcript levels did not differ between HUVECs treated with high glucose and related controls or between Tom22-deficient HUVECs and intact HUVECs (Supplementary Information, Figures S8A and S8B). Overexpression of Tom22 also did not significantly alter the mRNA levels of Mfn1 compared with those in negative controls in HUVECs (Figure S8C). These data suggested that the change in Mfn1 protein levels is posttranscriptionally regulated.

### 3.6. Reduction in Mfn1 Expression Increases Mitochondrial Fission and Cell Dysfunction

**Figure 8:**
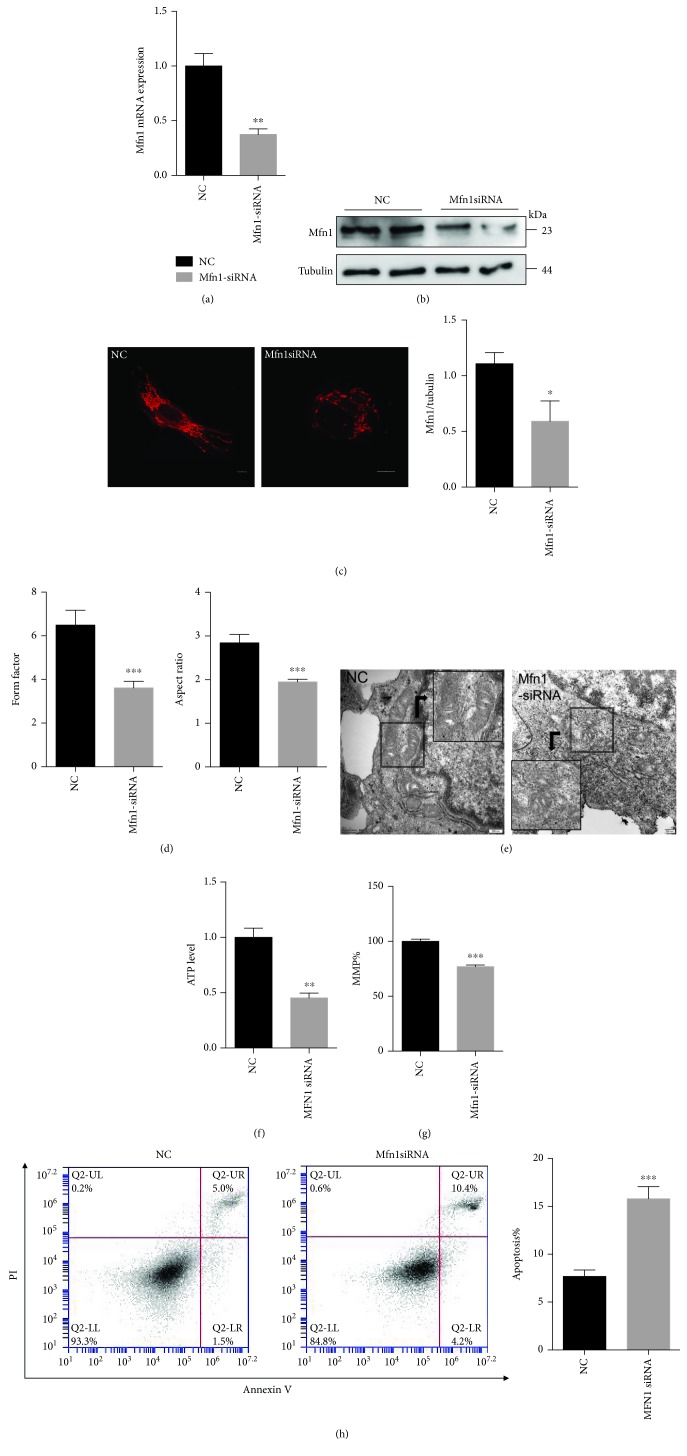
Downregulation of Mfn1 decreases mitochondrial fusion and induces mitochondrial dysfunction. HUVECs were transfected with Mfn1 siRNA to knock down the expression of Mfn1. NC represents the negative control. (a) The downregulation of Mfn1 was confirmed by real-time PCR (*n* = 3). (b) The expression of the Mfn1 protein was detected by Western blotting and quantification analysis (*n* = 3). (c) Representative confocal microscopy images of Mfn1 knockdown HUVECs stained with MitoTracker® Deep Red. Scale bar: 10 *μ*m. (d) Quantification of the form factor and aspect ratio of the mitochondrial networks (*n* = 4). (e) Representative images of mitochondria visualized by transmission electron microscopy. Mitochondria shortened in length in the Mfn1 knockdown group. Scale bar: 200 nm. (f) Total ATP level in HUVECs. (g) MMP was analyzed by JC1 staining and quantified based on fluorescence intensities (*n* = 3). (h) Apoptotic cells were detected by Annexin V/PI staining (*n* = 4). All data are presented as means ± SEMs (^∗^ *P* < 0.05, ^∗∗^ *P* < 0.01, and ^∗∗∗^ *P* < 0.001 vs. NC).

To determine whether the reduction in Mfn1 expression is critically responsible for the Tom22 deficiency-induced change in mitochondrial morphology and increase in cell apoptosis, we further tested whether the loss of Mfn1 function in HUVECs would produce a phenotype similar to that produced by Tom22 deficiency. Mfn1 siRNA was used to knock down the expression of Mfn1. The real-time PCR and western blot analyses confirmed that the expression of Mfn1 was downregulated (Figures 8(a) and 8(b)). HUVECs in the Mfn1 knockdown group exhibited increased mitochondrial fragmentation, while the negative-control HUVECs contained integrated networks and elongated mitochondrial tubules (Figure 8(c)). In addition, the average mitochondrial FF and AR values decreased significantly after Mfn1 knockdown (Figure 8(d)). The mitochondrial ultrastructure also showed more fragmentation in the Mfn1 knockdown group than in the control group (Figure 8(e)). Furthermore, the decreasing expression of Mfn1 led to reduced mRNA levels and protein levels of NDUFS1 and SDHB and did not affect the levels of mtCOx2 (Figures S4A and S4C). The knockdown of Mfn1 decreased the ATP production (Figure 8(f)) and MMP (Figure 8(g)) in HUVECs. The Annexin V/PI staining result demonstrated a substantial increase in cell apoptosis in the Mfn1 knockdown group compared with that in the control group (Figure 8(h)). Mitochondrial volume was reduced when Mfn1 was downregulated (Figure S4B).

### 3.7. Overexpression of Mfn1 Rescues Pathological Mitochondrial Dynamics and Dysfunction of HUVECs Induced by High Glucose

**Figure 9:**
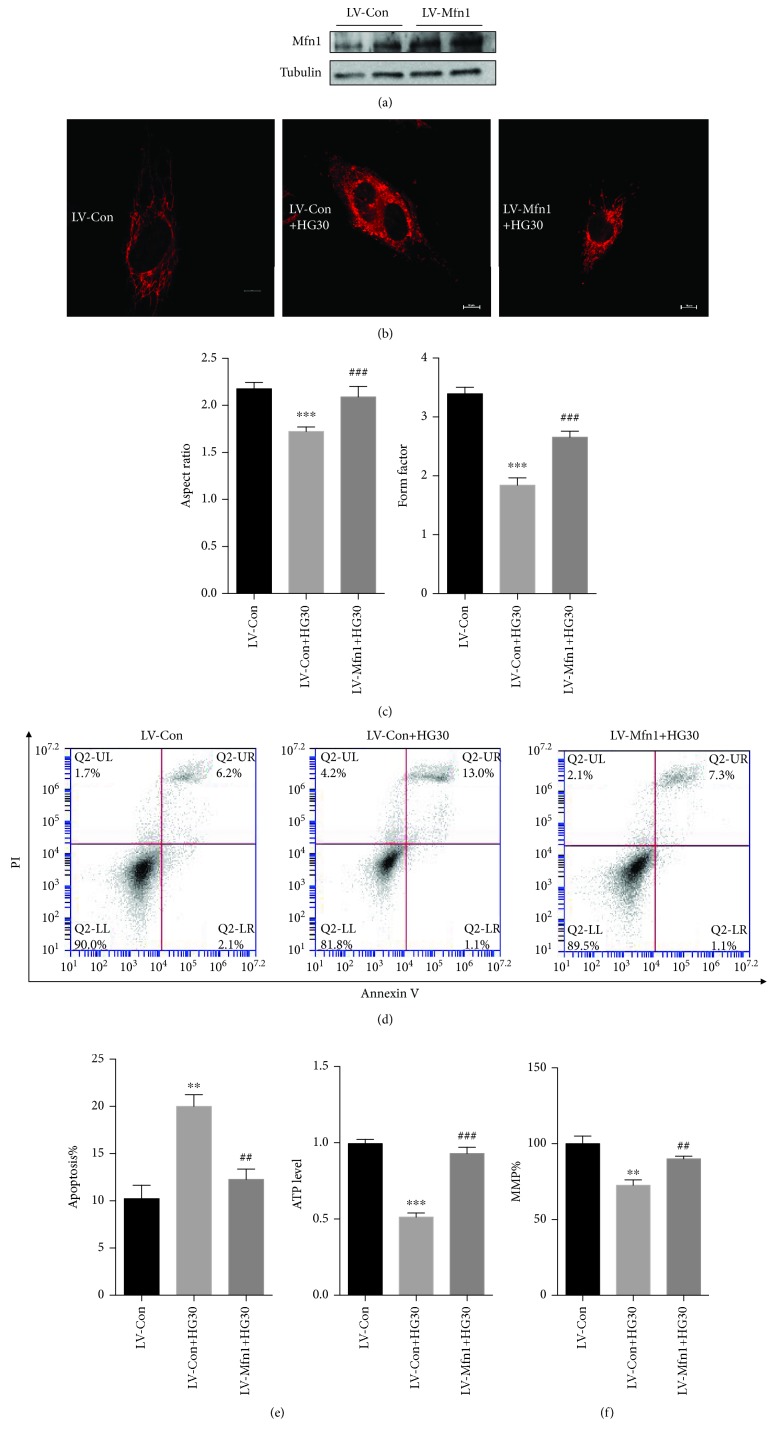
Overexpression of Mfn1 could protect against pathological mitochondrial dynamics and dysfunction of HUVECs induced by high glucose. HUVECs were transfected with LV-Mfn1 vectors that expressed Mfn1. The LV-Con vector was used as a vehicle control. (a) Western blot analysis was conducted to evaluate the overexpression efficiency of LV-Mfn1 (*n* = 3). (b) Representative confocal microscopy images of HUVECs stained with MitoTracker® Deep Red. Scale bar: 10 *μ*m. (c) Quantification of the form factor and aspect ratio of the mitochondrial networks (*n* = 4). (d) Apoptotic cells were detected by Annexin V/PI staining (*n* = 3). (e) Total ATP level in HUVECs (*n* = 4). (f) The MMP was analyzed by JC1 staining and quantified based on fluorescence intensities (*n* = 3). All data are presented as means ± SEMs (^∗^ *P* < 0.05, ^∗∗^ *P* < 0.01, and ^∗∗∗^ *P* < 0.001 vs. LV-Con; ^#^ *P* < 0.05, ^##^ *P* < 0.01, and ^###^ *P* < 0.001 vs. LV-Con+HG30).

The lentiviral vector-carrying gene Mfn1 was successfully constructed and used to transfect HUVECs. Western blot analyses confirmed the overexpression of Mfn1 (Figure 9(a) and Figure S5B). The mitochondria exhibited short rods and round fragments in the LV-Con+HG30 group. In addition, the mitochondria in the LV-Mfn1+HG30 group appeared as long tubular networks, which indicated that the increased expression of Mfn1 is sufficient to abolish the pathological mitochondrial dynamics induced by high glucose (Figure 9(b)). The mitochondria in the LV-Mfn1+HG30 group exhibited a higher FF and AR than that in the LV-Con+HG30 group (Figure 9(c)). The overexpression of Mfn1 restored the mRNA levels and protein levels of NDUFS1 and SDHB, which were decreased by high glucose and did not affect the levels of mtCOx2 (Figure S5A and Figure S5C). Furthermore, the ATP assay results showed that increased expression of Mfn1 improved the injury to the respiratory function of mitochondria caused by high glucose (Figure 9(d)), while overexpression of Mfn1 significantly alleviated the high glucose-induced loss of MMP and increase in apoptosis (Figures 9(e) and 9(f)). Increasing the expression of Mfn1 also improved the decreased mitochondrial volume and the increased ROS levels caused by high glucose (Figures S5D and S5E).

### 3.8. Downregulation of Drp1 Alone Cannot Rescue Completely Mitochondrial Defects Induced by High Glucose in HUVECs

Studies have reported that Drp1 plays a critical role in hyperglycemia induced mitochondria fragmentation; thus, we further investigate the role of Drp1 in this study. We used siRNA to knock down the expression of Drp1, which was confirmed by western blotting (Figure S6A). The level of p-Drp1 also significantly decreased in the Drp1 knockdown group, and knocking down the expression of Drp1 did not affect the level of Tom22 (Figure S6A). The mitochondria appeared as short fragments in the NC+HG30 group, and the mitochondria morphology in the Drp1 siRNA+HG30 group exhibited improved (Figure S6B). The mitochondria in the Drp1 siRNA+HG30 group exhibited a higher FF than did the mitochondria in the NC+HG30 group, but there was no significant difference in AR between the two groups (Figure S6C). The MMP was partially reversed by the downregulation of Drp1 under the high-glucose condition, but did not have significant difference between the Drp1 siRNA+HG30 group and the NC+HG30 group (Figure S6D).

## 4. Discussion

Studies have shown a distinct biological role of Tom22 in cellular function, but its functions in diabetes and endothelial cells remain unknown. Mitochondrial dynamics are recognized to play critical roles in mitochondrial functions, including development, apoptosis, and the functional complementation of mitochondrial DNA (mtDNA) mutations by content mixing [21, 27–29]. Shifting the balance of mitochondrial dynamics toward fission enhances the susceptibility to mitochondrial dysfunction, oxidative stress, and cell apoptosis [30]. The OXPHOS process is responsible for the production of ATP in mitochondria. There is evidence of dysfunction of OXPHOS in certain tissues relevant to the pathogenesis of diabetes, including reports of reduced expression of the electron transport chain (ETC) genes in adipose tissue of female type 2 diabetes [31] and downregulation of expression of genes involved in OXPHOS in muscles of patients with type 2 diabetes and of high-risk individuals [32, 33].

In this study, we discovered that Tom22 expression is significantly reduced in endothelial cells in a glucose dose-dependent and time-dependent manner. HUVEC exposure to high glucose induces a decrease in mitochondrial fusion and in gene expression of OXPHOS, which results in a reduction in both ATP production and MMP and an increase in ROS and apoptosis. Moreover, our results show that Tom22 knockdown decreased mfn1 and increased Fis1, Mff, and p-Drp1, leading to a shift in the balance of mitochondrial dynamics toward fission. Downregulation of Tom22 also results in reduced NDUFS1 and SDHB, which causes the OXPHOS process to be impaired. These results indicate that Tom22 mediates HUVEC dysfunction through the regulation of mitochondrial dynamics and OXPHOS processes and that Tom22 downregulation alone could mimic the injury phenotype of HUVECs induced by high glucose. To confirm this association, we overexpressed Tom22 in endothelial cells by lentiviral transduction. The overexpression of Tom22 maintains mitochondrial function and cell survival by reversing the high-glucose-induced changes in the expression of proteins regulating mitochondrial dynamics and OXPHOS. Mitochondrial dynamics and OXPHOS are both key processes of cell metabolism. We demonstrate that upregulation of Tom22 may protect HUVECs against high glucose stress by restoring the balance of mitochondrial dynamics and OXPHOS.

In addition, the mRNA levels of Tom22 significantly decreased at 15 mM glucose for 48 hr or at 30 mM glucose for 24 hr, which decreased more pronounced at 30 mM glucose for 48 hr. However, the protein levels of Tom22 decreased at some extent, but did not change significantly at 15 mM glucose for 48 hr or at 30 mM glucose for 24 hr. It was just decreased significantly at 30 mM glucose for 48 hr. Therefore, we choose 30 mM and 48 hr for this study. The mRNA levels of Tom22 significantly decreased at 15 mM glucose for 48 hr, and the protein levels of Tom22 also decreased to some extent at 15 mM glucose, which may be sufficient to reduce mitochondrial fusion and OXPHOS partially, in turn resulting in MMP and ATP production defects. These changes were more pronounced when Tom22 was decreased significantly at 30 mM glucose. Besides, alteration of mitochondrial dynamics and OXPHOS may both contribute to the changes of mitochondrial volume, which also explains the increased levels of ROS. The mtDNA copy number did not change significantly after high glucose treatment. It is possible that the change in mtDNA copy number is a delayed response of these processes and had not yet occurred at the early stage studied here.

Furthermore, our results showed that the mRNA levels of Tom22 were reduced in endothelial cells exposed to high glucose, indicating a defect in the transcriptional regulation of Tom22. Previous studies revealed that casein kinase 1 (CK1), casein kinase 2 (CK2), and protein kinase A (PKA) could regulate the import and assembly of Tom22. Thus, transcriptional and posttranscriptional regulation may both account for the reduction in Tom22 in endothelial cells treated with high glucose. Moreover, AMP-activated protein kinase (AMPK) acts as an energetic stress sensor that is widely involved in the regulation of cellular metabolism [34]. Response of AMPK/PGC-1*α* to glucose may also be associated with the alteration of Tom22. The specific upstream signaling of Tom22 requires further research. We found that only Tom22 decreased, whereas the other members, including Tom20, Tom40, and Tom70, did not change significantly upon exposure to high glucose. Therefore, we only explored the role of Tom22 in this study. Li et al. revealed that Tom70 acts as a molecular switch that functions in hypertrophic stresses and mitochondrial responses to determine pathological cardiac hypertrophy [10]. Chronic contractile activity modulates biogenesis of the mitochondrial Tom40 channel in skeletal muscle [35]. Thus, members of this complex may have different responses to different stresses.

Mfn1 is a GTPase protein located in the outer mitochondrial membrane and is essential for mitochondrial fusion [36, 37]. Considerable evidence has highlighted Mfn1 as a downstream effector of the Tom22-dependent pathway underlying the high glucose-induced dysfunction of endothelial cells. First, high glucose reduced the expression of Tom22 and Mfn1 protein and decreased mitochondrial fusion and OXPHOS. Second, the reduction in Tom22 expression reduced mitochondrial fusion and OXPHOS in HUVECs. Third, CO-IP showed that Tom22 directly binds to mfn1. Fourth, the phenotypes associated with mfn1 deficiency closely mimicked the phenotypes caused by Tom22 reduction. Finally, overexpression of Mfn1 could rescue pathological mitochondrial dynamics, proteins of OXPHOS, and dysfunction of HUVECs induced by high glucose.

As to the mechanisms that may explain the effect of high glucose on OXPHOS expression, our results showed that downregulation of Tom22 significantly decreased the mRNA levels and protein levels of NDUFS1 and SDHB and did not affect the levels of mtCOx2. Overexpression of the reduced Tom22 induced by high glucose could reverse the changes to the mRNA and protein levels of OXPHOS. Downregulation of Mfn1 reverses the changes in the mRNA and protein levels of OXPHOS. Increasing the expression of Mfn1 also could restore the levels of SDHB and NDUFS1 under high-glucose conditions. In addition, a recent study reported that Mfn2 upregulates fuel oxidation through expression of the OXPHOS system [38]. Therefore, we suspect that Mfn1 may induce signals that affect the expression of nuclear genes encoding OXPHOS subunits. The discrepancy between nuclear and mitochondrial DNA expression may explain the appearance of ROS. The hypothesis is currently under investigation.

In addition, the p-Drp1 response to alteration of Tom22 induced by high glucose was investigated. Downregulation or overexpression of Tom22 can modulate the expression of p-Drp1. A reduced level of Drp1 could not rescue completely mitochondrial defects that were induced by high glucose. Therefore, Tom22 was the upstream of the effector of Drp1 and p-Drp1. Drp1 was also partially involved in the Tom22-induced alteration of mitochondrial dynamics, but it alone cannot affect the expression of Tom22. Opa1 was cleaved from long (L-Opa1) to short (S-Opa1) forms. L-Opa1 is required for mitochondrial fusion, but accumulation of S-Opa1 in excess accelerates fission [39, 40]. L-Opa1/S-Opa1 did not change significantly in HUVECs under the high-glucose condition. Maybe there are some differences in the roles of genes that involved mitochondrial dynamics in different cells in response to stresses.

There are several open questions worth future study. First, mitochondrial fusion and fission undergo changes under hyperglycemic conditions in a variety of cell types, including endothelial cells [20, 21], pancreatic *β* cells [41, 42], and skeletal muscle cells [43]. In addition, Tom22 is widespread in all organs and cells of humans. Thus, it is meaningful to investigate the specific molecular mechanisms and responses of other cells in future studies. Second, glucose transporter-1 (GLUT-1) is the major glucose-transporting protein of endothelial cells [44]. We also can explore the effects caused by blocking glucose entry to the cells in the near future to broaden the horizon of our work. Third, it has been reported that metformin treatments markedly reduced mitochondrial fragmentation, mitigated mitochondrial-derived superoxide release, and inhibited vascular inflammation [45]. Schisano et al. suggested that liraglutide (a GLP-1 analogue) may prevent high-glucose-induced mitochondrial fragmentation and apoptosis in human endothelial cells by inducing mitochondrial fusion processes [46]. Therefore, these drugs may have effects on regulating Tom22 and mitochondrial dynamics in HUVECs. It is worthwhile to identify protective drugs and potential drug targets. Fourth, it would also be helpful to assess the numbers of assembled complexes using native PAGE to determine whether there are less active complexes available upon glucose exposure, and the mechanisms by which Mfn1 regulates the OXPHOS process should be further elucidated. We will design studies to test these hypotheses in the near future.

In conclusion, the present study identified Tom22 as a critical regulator of the high-glucose-induced dysfunction and apoptosis of endothelial cells. Mechanistically, Tom22 governs mitochondrial dynamics and OXPHOS to dramatically abolish the insults induced by high glucose. Moreover, Mfn1 is a downstream effector of Tom22 that modulates mitochondrial dynamics and OXPHOS. The identification of a correlation between Tom complex defects and endothelial dysfunction may provide a novel therapeutic target for the prevention and management of atherosclerosis and diabetic cardiovascular disease.

## Data Availability

The data used to support the findings of this study are available from the corresponding author upon request.

## Supplementary Materials

Supplementary Materials Figure S1: related to Figure 2. Expression of Tom complex subunit, OXPHOS, and mitochondrial mass in HUVECs exposed to 30 mmol/l D-glucose for 48 hr. Figure S2: related to Figure 4. Expression of Tom complex subunit and gene of OXPHOS and mitochondrial mass in NC and Tom22 knockdown group. Figure S3: related to Figure 6. Expression of Tom complex subunit and gene of OXPHOS and mitochondrial mass in the control and Tom22OE group exposed to 30 mM glucose. Figure S4: related to Figure 8. Expression of gene of OXPHOS and mitochondrial volume in NC and Mfn1 knockdown group. Figure S5: related to Figure 9. Expression of gene of OXPHOS and mitochondrial volume in the control and Mfn1OE groups exposed to 30 mM glucose. Figure S6: downregulation of Drp1 alone cannot rescue completely mitochondrial defects induced by high glucose in HUVECs. Figure S7: the effect of different siRNA to knock down the expression of Tom22, Mfn1, and Drp1. Figure S8: the mRNA levels of Mfn1 assessed by real-time PCR.
