# Supplementary material for: Impaired Mitochondrial Fusion and Oxidative Phosphorylation Triggered by High Glucose Is Mediated by Tom22 in Endothelial Cells
Source: Oxid Med Cell Longev. 2019 May 19;2019:4508762. doi: 10.1155/2019/4508762 (PMC6545771; doi:10.1155/2019/4508762)
Supplement: Supplementary Materials — Figure S1: related to Figure 2. Expression of Tom complex subunit, OXPHOS, and mitochondrial mass in HUVECs exposed to 30 mmol/l D-glucose for 48 hr. Figure S2: related to Figure 4. Expression of Tom complex subunit and gene of OXPHOS and mitochondrial mass in NC and Tom22 knockdown group. Figure S3: related to Figure 6. Expression of Tom complex subunit and gene of OXPHOS and mitochondrial mass in the control and Tom22OE group exposed to 30 mM glucose. Figure S4: related to Figure 8. Expression of gene of OXPHOS and mitochondrial volume in NC and Mfn1 knockdown group. Figure S5: related to Figure 9. Expression of gene of OXPHOS and mitochondrial volume in the control and Mfn1OE groups exposed to 30 mM glucose. Figure S6: downregulation of Drp1 alone cannot rescue completely mitochondrial defects induced by high glucose in HUVECs. Figure S7: the effect of different siRNA to knock down the expression of Tom22, Mfn1, and Drp1. Figure S8: the mRNA levels of Mfn1 assessed by real-time PCR. [file 4508762.f1.pdf]

We also measured the protein expression of Tom20, Tom40 and Tom70. High glucose didn't affect the protein levels of Tom20, Tom40 and Tom70 significantly(Fig.S1A). Western blotting revealed a significant increase in the levels of pDrp1 in HUVECs after treatment with high glucose (Fig.S1B). The levels of L-Opa1/S-Opa1 were unchanged (Fig.S1C). The mRNA levels of NDUFS1 and SDHB was significantly decreased in HUVECs exposed to high glucose (Fig. S1D). High glucose did not affect the mRNA levels of mtCOx2 in HUVECs (Fig. S1D). The mtDNA copy number was not changed significantly in HUVECs exposed to high glucose (30 mmol/l) compared with that under normal conditions (Fig. S1E). Mitochondrial volume was significantly decreased in HUVECs exposed to high glucose (Fig. S1F). We found that high glucose significantly increased the ROS levels of HUVECs (Fig. S1G).

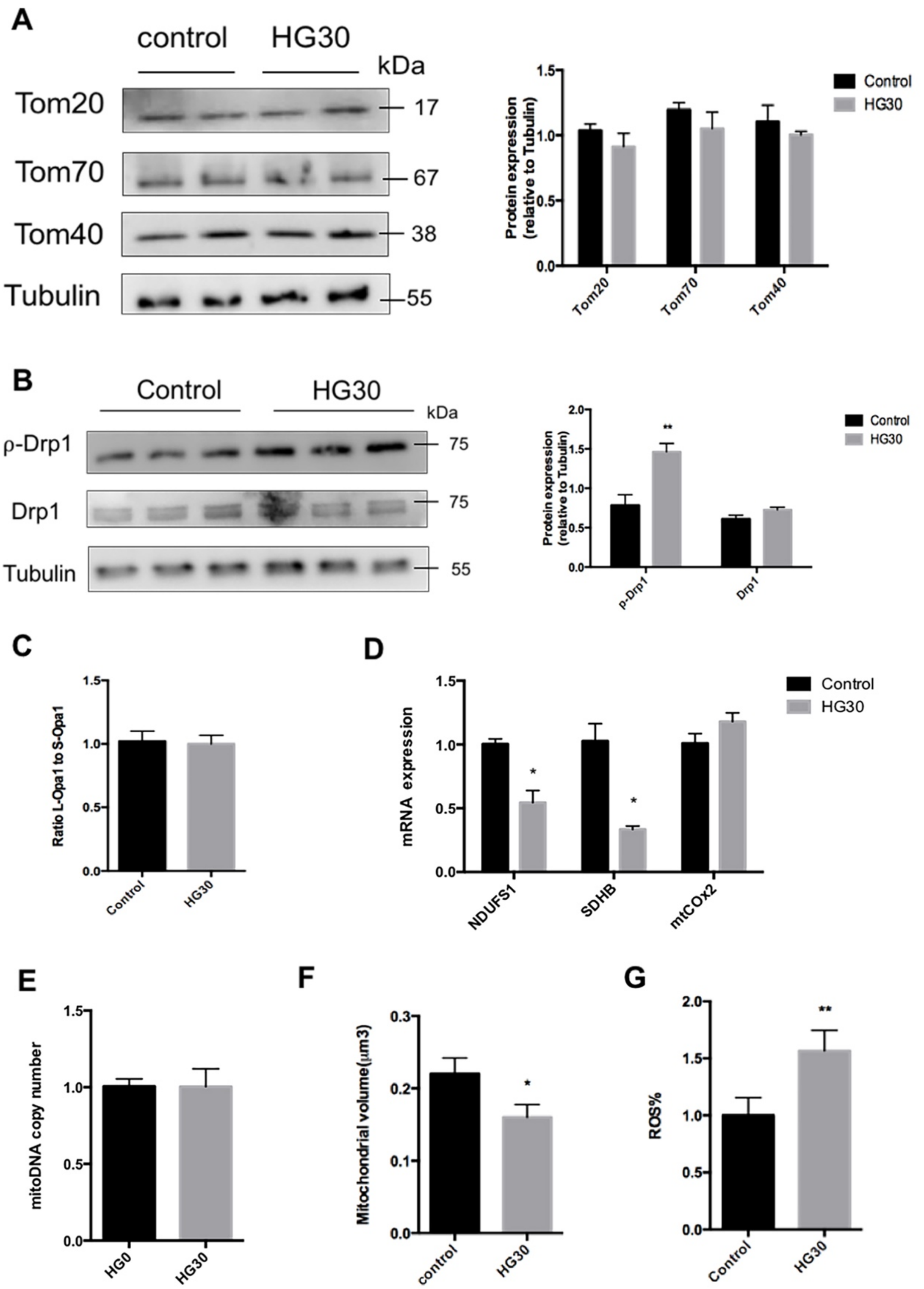

Figure S1. Related to Figure 2. Expression of Tom complexes subunit, OXPHOS and mitochondrial mass in HUVECs exposed to 30 mmol/l D-glucose for 48 hr
(A) Representative Western blot and quantification analysis of the Tom20, Tom40 and Tom70 protein level in HUVECs exposed to 30 mmol/l D-glucose for 48 hr(n=3).

(B) Representative Western blot and quantification analysis of the p-Drp1 protein level in HUVECs exposed to 30 mmol/l D-glucose for 48 hr(n=3).
(C) Quantification analysis of L-Opa1/S-Opa1 of Fig.2A(n=3).
(D) Real-time PCR analysis of OXPHOS gene in HUVECs exposed to 30 mmol/l D-glucose for 48 hr(n=3).
(E) Real-time PCR analysis of mitochondrial copy number in HUVECs exposed to 30 mmol/l D-glucose for 48hr(n=3).
(F) Mitochondrial volume per cell in HUVECs exposed to 15mM or 30mM D-glucose for 48 hr(n=3).
(G) The ROS levels of HUVECs exposed to 30 mmol/l D-glucose for 48 hr(n=3).
(* $P < 0.05$; ** $P < 0.01$ vs. Control;) Control: normal culture medium; HG15: D-glucose 15 mmol/l; HG30: D-glucose 30 mmol/l. All data are presented as the mean ± SEMs.

There was no significant difference in protein levels of Tom20, Tom40 and Tom70 between the Tom22 knockdown group and the NC group (Fig. S2A). The expression of the pDrp1 proteins was significantly higher in the Tom22 knockdown cells than in the control cells(Fig.S2B). Furthermore, the levels of L-Opa1/S-Opa1 were unchanged (Fig. S2C). Downregulation of Tom22 significantly decreased the mRNA levels of NDUFS1 and SDHB (Fig. S2D). There was no significant difference in expression of mtCOx2 between the two groups (Fig. S2D). Knockdown of Tom22 did not significantly affect the mtDNA copy number (Fig. S2E). Mitochondrial volume was reduced when Tom22 was downregulation (Fig. S2F).

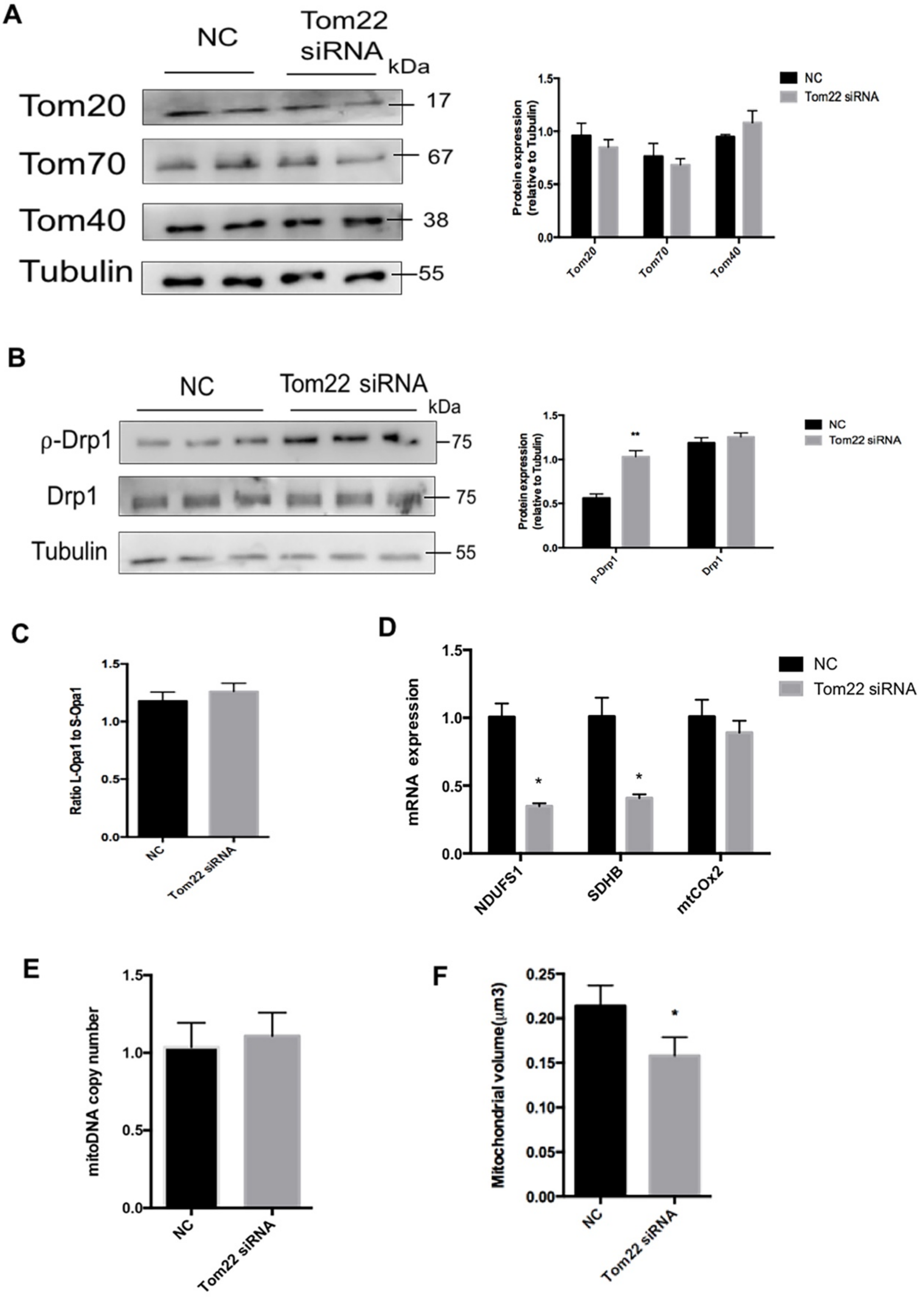

Figure S2. Related to Figure 4. Expression of Tom complexes subunit and gene of OXPHOS and mitochondrial mass in NC and Tom22 knockdown group

(A) Representative Western blot and quantification analysis of the Tom20, Tom40 and Tom70 protein level in HUVECs (n=3).

(B) Representative Western blot and quantification analysis of the p-Drp1 protein level in HUVECs(n=3).

(C) Quantification analysis of L-Opa1/S-Opa1 of Fig.4A(n=3).

(D) Real-time PCR analysis of OXPHOS gene in NC and Tom22 knockdown group (n=3).

(E) Real-time PCR analysis of mitochondrial copy number in HUVECs(n=3).

(F) Mitochondrial volume per cell of HUVECs(n=3).

(* $P < 0.05$; ** $P < 0.01$ vs.NC) NC: HUVECs transfected with negative control siRNA; Tom22 siRNA: HUVECs transfected with Tom22 siRNA to knock down the expression of Tom22. All data are presented as the means ± SEMs.

The protein levels of Tom20, Tom40 and Tom70 did not change significantly when TOM22 was overexpressed(Fig.S3A). Tom22 overexpression significantly decreased the levels of p-Drp1 that were increased under high-glucose conditions (Fig. S3B). Quantification of western blotting of the Fig.5B (Fig. S3C). There was no significant difference in protein expression of L-Opa1/S-Opa1 between the LV-Tom22 and LV-Con groups under the high-glucose condition (Fig. S3D). In addition, mRNA levels of NDUFS1 and SDHB were significantly increased in LV-Tom22 group under high glucose compared with their levels in the LV-Con group (Fig. S3E). There was no significant difference in mtDNA copy number between the LV-Con group and the LV-Tom22 group (Fig. S3F). Mitochondrial volume was significantly increased in LV-Tom22 group than that of LV-Con group under the 30mM glucose condition (Fig. S3G). Overexpression of Tom22 could restore the ROS levels that were increased by high glucose (Fig. S3H).

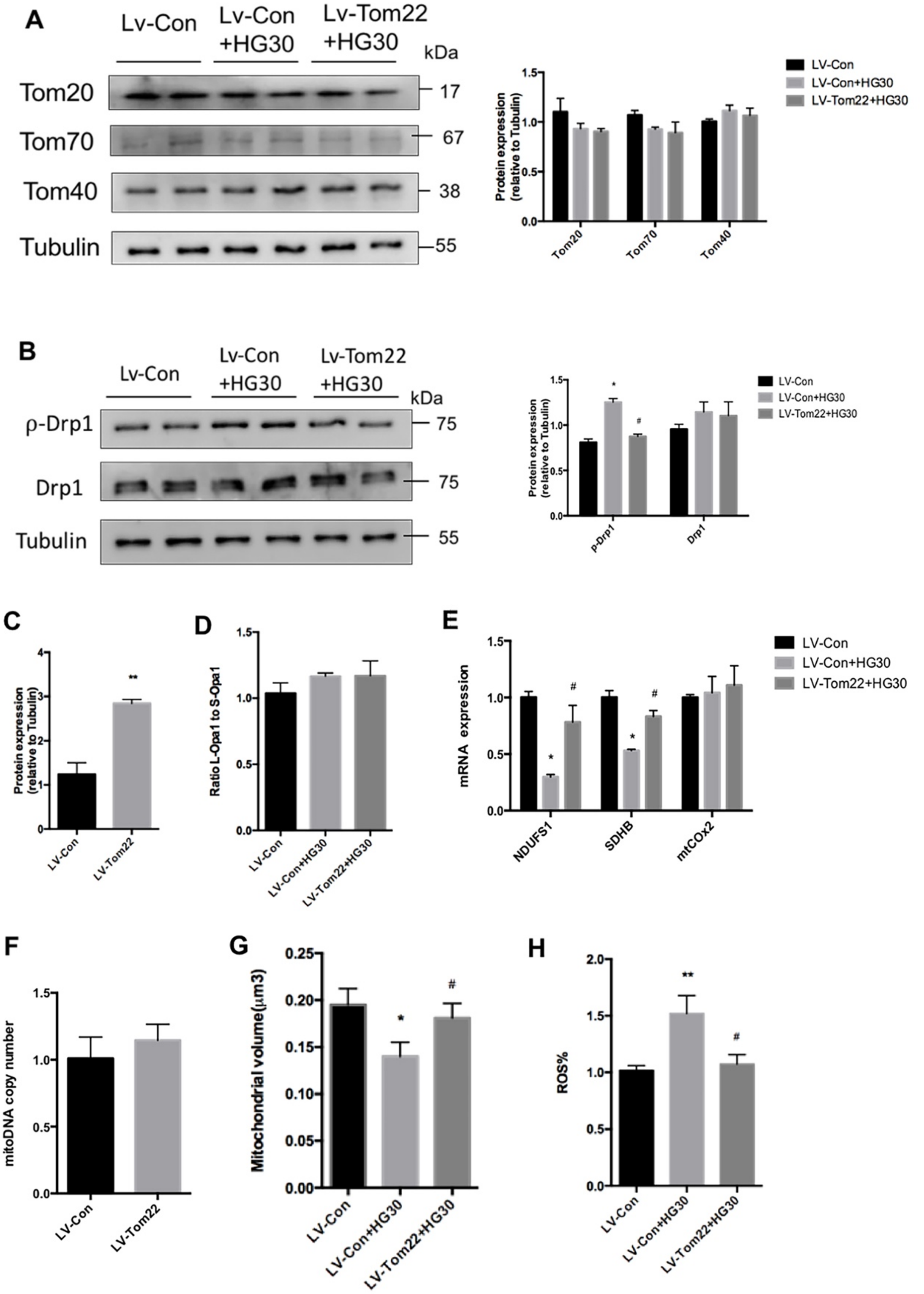

Figure S3. **Related to Figure 6. Expression of Tom complexes subunit and gene of OXPHOS and mitochondrial mass in control and Tom22OE group exposed to 30mM glucose.** (HUVECs were transduced with LV-Tom22 vectors that expressed Tom22. The LV-Con vector was used as a vehicle control)

(A) Representative Western blot and quantification analysis of the Tom20, Tom40 and Tom70 protein level in HUVECs (n=3).
(B) Representative Western blot and quantification analysis of the p-Drp1 protein level in HUVECs (n=3).
(C) Quantification analysis of Tom22 of Fig.5B(n=3).
(D) Quantification analysis of L-Opa1/S-Opa1 of Fig.6A(n=3).
(E) Real-time PCR analysis of OXPHOS gene in HUVECs (n=3).
(F) Real-time PCR analysis of mitochondrial copy number in HUVECs (n=3).
(G) Mitochondrial volume per cell in HUVECs (n=3).
(H) The ROS levels of HUVECs(n=3).
All data are presented as the means ± SEMs (* $P < 0.05$, **$P < 0.01$ vs. LV-Con; # $P < 0.05$, ## $P < 0.01$ vs. LV-Con+HG30).

The decreasing expression of Mfn1 led to reduced mRNA levels and protein levels of NDUFS1 and SDHB and did not affect the levels of mtCOx2 (Fig.S4A and Fig.S4C). Mitochondrial volume was reduced when Mfn1 was downregulation (Fig. S4B).

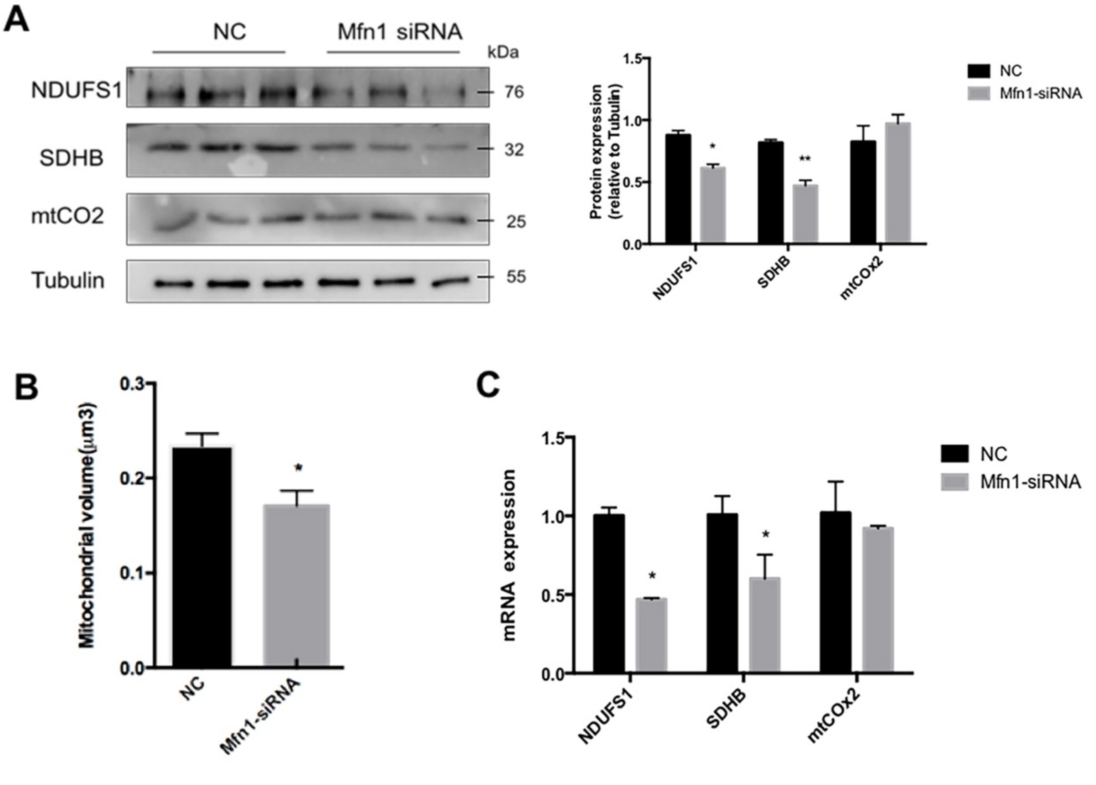

Figure S4. Related to Figure 8. Expression of gene of OXPHOS and mitochondrial volume in NC and Mfn1 knockdown group
(HUVECs were transfected with Mfn1 siRNA to knock down the expression of Mfn1. NC represents the negative control.)

(A) Representative Western blot and quantification analysis of the level of proteins that involve in OXPHOS(n=3).
(B) Mitochondrial volume per cell of HUVECs(n=3).
(C) Real-time PCR analysis of OXPHOS gene in NC and Mfn1 knockdown group (n=3).
All data are presented as the means ± SEMs (* $P < 0.05$, ** $P < 0.01$ vs. NC).

The overexpression of Mfn1 restored the mRNA levels and protein levels of NDUFS1 and SDHB, which were decreased by high glucose and did not affect the levels of mtCOx2 (Fig.S5A and Fig.S5C). Quantification of western blotting of the Fig.9A(Fig.S5B) Increasing the expression of Mfn1 also improved the decreased mitochondrial volume and the increased ROS levels caused by high glucose (Fig.S5D and Fig.S5E).

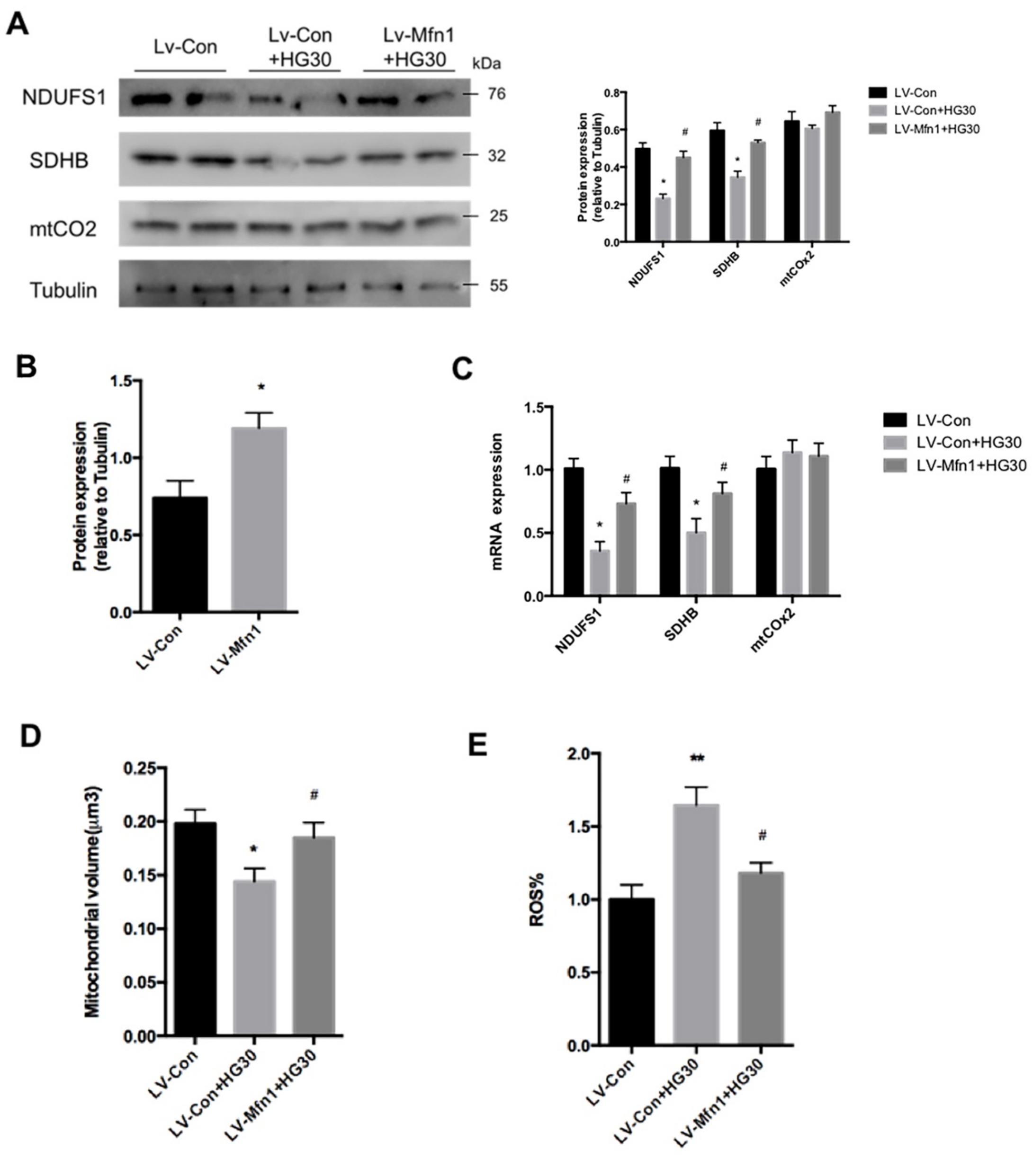

Figure.S5 Related to Figure 9. Expression of gene of OXPHOS and mitochondrial volume in control and Mfn1OE group exposed to 30mM glucose (HUVECs were transfected with LV-Mfn1 vectors that expressed Mfn1. The LV-Con vector was used as a vehicle control.)

(A) Representative Western blot and quantification analysis of the Tom20, Tom40 and Tom70 protein level in HUVECs (n=3).

(B) Quantification analysis of Tom22 of Fig.9A(n=3).

(C) Real-time PCR analysis of OXPHOS gene in HUVECs (n=3).

(D) Mitochondrial volume per cell in HUVECs (n=3).

(E) The ROS levels of HUVECs(n=3).

All data are presented as the means ± SEMs. (* $P < 0.05$, ** $P < 0.01$ vs. LV-Con; # $P < 0.05$, ## $P < 0.01$ vs. LV-Con+HG30).

We used siRNA to knock down the expression of Drp1, which was confirmed by western blotting(Fig.S6A). The level of p-Drp1 also significantly decreased in the Drp1 knockdown group, and knocking down the expression of Drp1 did not affect the protein levels of Tom22 (Fig.S6A). The mitochondria appeared as short fragments in the NC+HG30 group, and the mitochondria morphology in the Drp1 siRNA+HG30 group exhibited improved (Fig.S6B). The mitochondria in the Drp1 siRNA+HG30 group exhibited a higher FF than did the mitochondria in the NC+HG30 group, but there is no significant difference in AR between the two group(Fig.S6C). The MMP was partially reversed by the downregulation of Drp1 under the high-glucose condition, but did not have significant difference between Drp1 siRNA+HG30 group and NC+HG30 group(Fig.S6D).

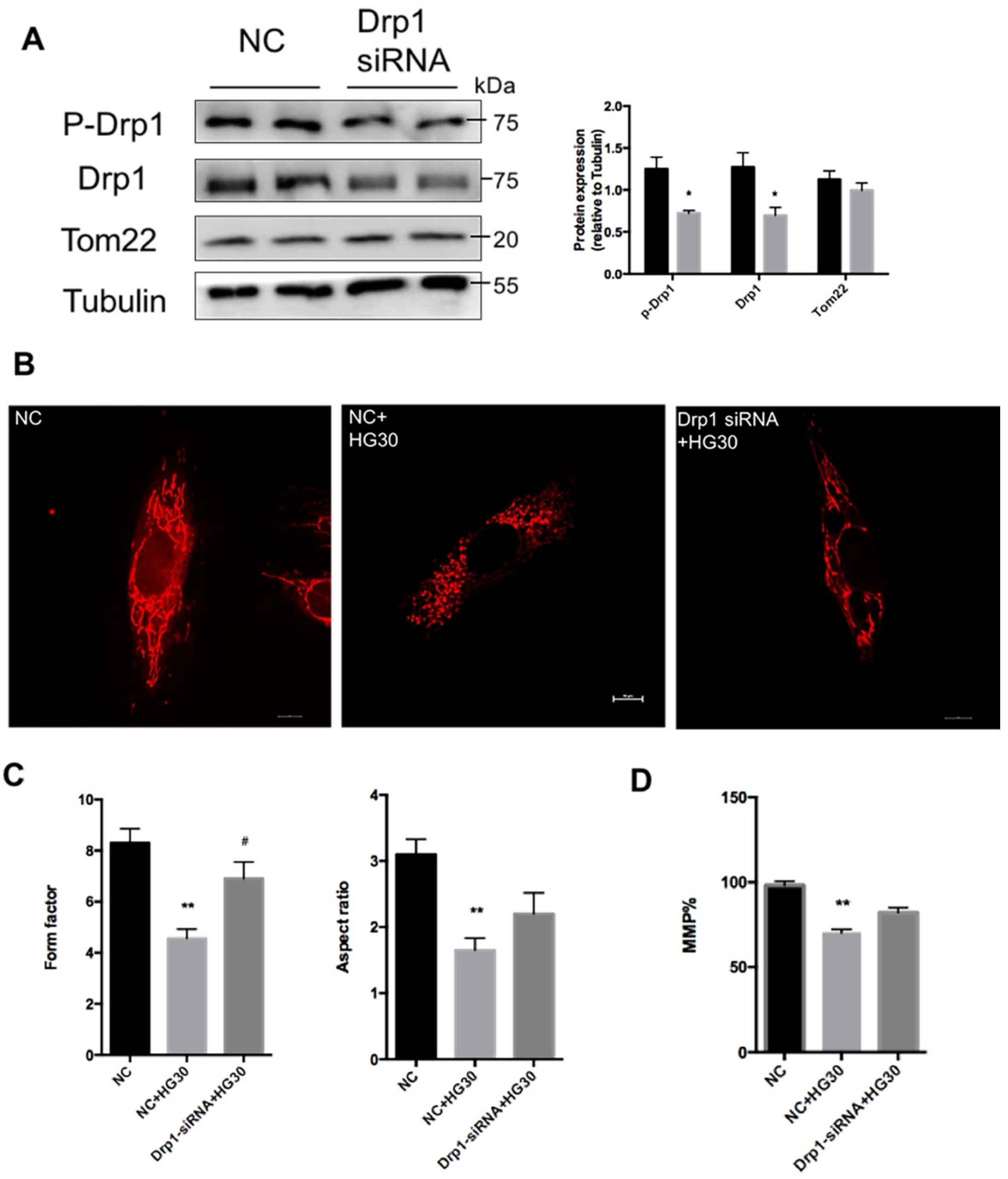

Figure.S6 Downregulation of Drp1 alone can't rescue completely mitochondrial defects induced by high glucose in HUVECs (HUVECs were transfected with Drp1 siRNA to knock down the expression of Drp1. NC represents the negative control.)

(A) Representative Western blot and quantification analysis in NC and Drp1 knockdown group (n=3).

(B) Cells were stained with MitoTracker. Scale bar: 10 µm. (n=3).

(C) Quantification of the form factor and aspect ratio of the mitochondrial networks (n=3).

(D) The MMP was analyzed by JC1 staining and quantified based on fluorescence intensities(n=3).

All data are presented as the means ± SEMs. (* $P < 0.05$, **$P < 0.01$ vs. NC; # $P < 0.05$, ## $P < 0.01$ vs. NC+HG30).

We used three pairs of siRNA to knockdown the expression of Tom22. The effect of siRNA 342 was most significant and was used for subsequent experiments.
Three pairs of siRNA were used to knockdown the expression of Mfn1. The effect of siRNA 982 was most obvious and was used for subsequent experiments.
We used three pairs of siRNA to knockdown the expression of Drp1, which was confirmed by western blot. The effect of siRNA 2083 was most obvious and was used for subsequent experiments.

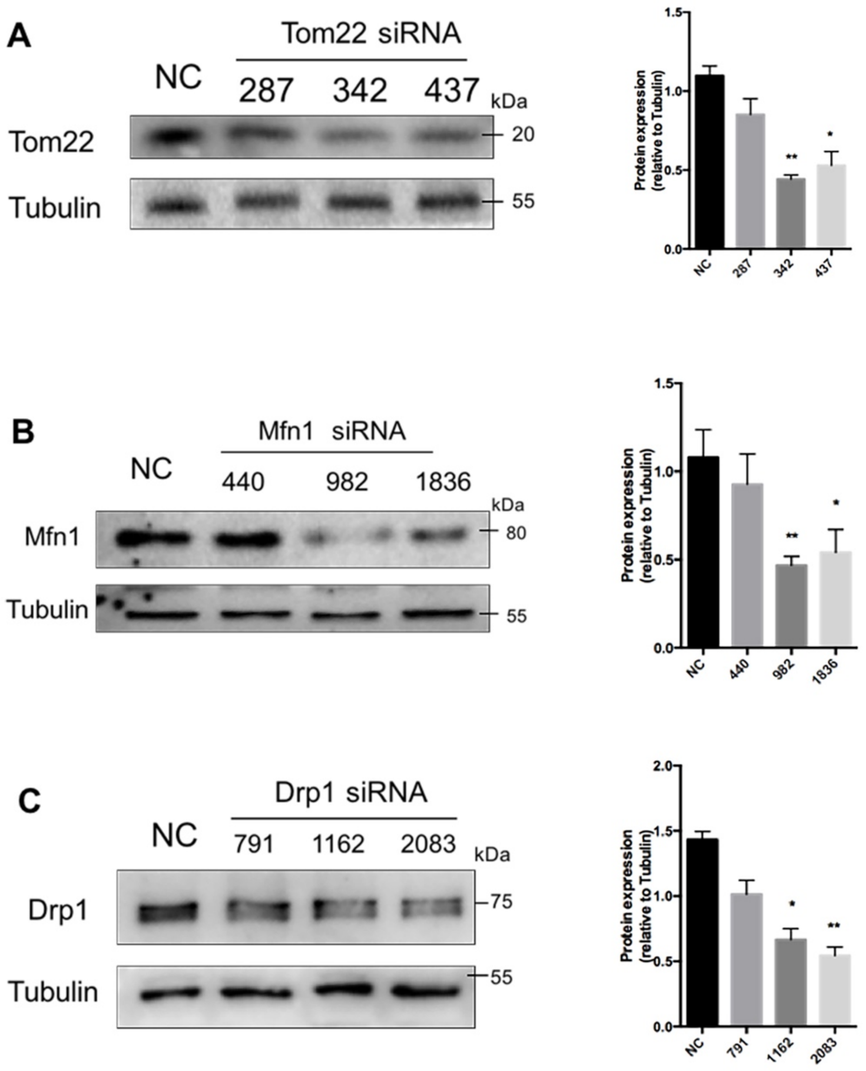

Figure S7. The effect of different siRNA to knockdown the expression of Tom22, Mfn1 and Drp1.

(A)The effect of three pairs of siRNA to knockdown the expression of Tom22 (n=3).

(B) The effect of three pairs of siRNA to knockdown the expression of Mfn1 (n=3).
(C) The effect of three pairs of siRNA to knockdown the expression of Drp1 (n=3).
All data are presented as the means ± SEMs. (* P < 0.05, **P < 0.01 vs. NC)

we quantified the mRNA levels of Mfn1 via real-time PCR. We observed that the Mfn1 transcript levels did not differ between HUVECs treated with high glucose and related controls or between Tom22-deficient HUVECs and intact HUVECs (Supplementary information, Fig. S8A and S8B). Overexpression of Tom22 also did not significantly alter the mRNA levels of Mfn1 compared with those in negative controls in HUVECs (Fig. S8C).

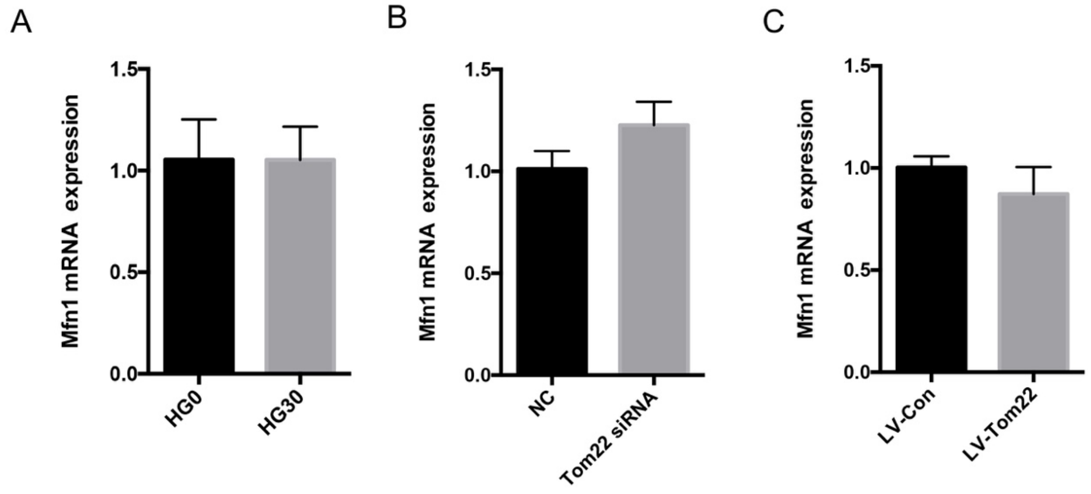

Figure S8. The mRNA levels of Mfn1 assessed by Real-time PCR
(A)The mRNA levels of Mfn1 in HUVECs exposed to 30 mmol/l D-glucose for 48hr(n=3).
(B)The mRNA levels of Mfn1 in NC and Tom22 knockdown group(n=3).
(C)The mRNA levels of Mfn1 in LV-Con group and LV-Tom22 group(n=3).
